# Valores de Normalidade para Ressonância Magnética Cardiovascular: Uma Revisão Analítico-Comparativa com Ênfase na População Brasileira

**DOI:** 10.36660/abc.20250862

**Published:** 2026-07-21

**Authors:** Jorge A. Torreão, Isabela Bispo Santos da Silva Costa, Antonildes N. Assunção-Jr, Ilan Gottlieb, Leonardo Sara da Silva, Tiago Senra, Tiago Augusto Magalhães, Marly Uellendahl, Hugo Bizetto Zampa, Gabriela Liberato, Walther Yoshiharu Ishikawa, Henrique Trad, Marcelo Hadlich, Otavio Coelho-Filho, Roberto Sasdelli, Juliano Lara Fernandes, Luiz Francisco Rodrigues de Ávila, Ibraim Masciarelli F. Pinto, Paulo R. Schvartzman, Carlos E. Rochitte

**Affiliations:** 1 Hospital das Clínicas da Faculdade de Medicina da Universidade de São Paulo Instituto do Coração São Paulo SP Brasil Instituto do Coração do Hospital das Clínicas da Faculdade de Medicina da Universidade de São Paulo, São Paulo, SP – Brasil; 2 Hospital Santa Izabel Salvador BA Brasil Hospital Santa Izabel – Santa Casa da Bahia, Salvador, BA – Brasil; 3 Diagnósticos da América S/A – DASA São Paulo SP Brasil Diagnósticos da América S/A – DASA, São Paulo, SP – Brasil; 4 Rede D’Or Regional Bahia Salvador BA Brasil Rede D’Or Regional Bahia, Salvador, BA – Brasil; 5 Universidade de São Paulo Instituto do Câncer do Estado de São Paulo São Paulo SP Brasil Universidade de São Paulo Instituto do Câncer do Estado de São Paulo, São Paulo, SP – Brasil; 6 Fonte Imagem Medicina Diagnóstica Rio de Janeiro RJ Brasil Fonte Imagem Medicina Diagnóstica, Rio de Janeiro, RJ – Brasil; 7 Clínica CDI PREMIUM Goiânia GO Brasil Clínica CDI PREMIUM, Goiânia, GO – Brasil; 8 Instituto Dante Pazzanese de Cardiologia São Paulo SP Brasil Instituto Dante Pazzanese de Cardiologia, São Paulo, SP – Brasil; 9 Hospital Sírio Libanês São Paulo SP Brasil Hospital Sírio Libanês, São Paulo, SP – Brasil; 10 Hospital Vera Cruz Campinas SP Brasil Hospital Vera Cruz, Campinas, SP – Brasil; 11 Complexo Hospital de Clínicas da Universidade Federal do Paraná Curitiba PR Brasil Complexo Hospital de Clínicas da Universidade Federal do Paraná (CHC-UFPR) – Cardiovascular CT/MR, Curitiba, PR – Brasil; 12 Hospital do Coração São Paulo SP Brasil Hospital do Coração (HCOR), São Paulo, SP – Brasil; 13 Universidade Federal de São Paulo São Paulo SP Brasil Universidade Federal de São Paulo (UNIFESP), São Paulo, SP – Brasil; 14 Hospitais São Luiz São Paulo SP Brasil Hospitais São Luiz, Rede D’Or, São Paulo, SP – Brasil; 15 Lotus Radiologia Ribeirão Preto SP Brasil Lotus Radiologia, Ribeirão Preto, SP – Brasil; 16 Instituto Nacional de Cardiologia Rio de Janeiro RJ Brasil Instituto Nacional de Cardiologia, Rio de Janeiro, RJ – Brasil; 17 Universidade Estadual de Campinas Campinas SP Brasil Universidade Estadual de Campinas, Campinas, SP – Brasil; 18 Einstein Hospital Israelita São Paulo SP Brasil Einstein Hospital Israelita, São Paulo, SP – Brasil; 19 Radiologia Clínica de Campinas Campinas SP Brasil Radiologia Clínica de Campinas, Campinas, SP – Brasil; 20 Instituto de Ensino e Pesquisa Jose Michel Kalaf Campinas SP Brasil Instituto de Ensino e Pesquisa Jose Michel Kalaf, Campinas, SP – Brasil; 21 Grupo Fleury São Paulo SP Brasil Grupo Fleury, São Paulo, SP – Brasil; 22 Hospital Moinhos de Vento Porto Alegre RS Brasil Hospital Moinhos de Vento, Porto Alegre, RS – Brasil

**Keywords:** Valores de Referência, Imageamento por Ressonância Magnética, Doenças Cardiovasculares

## Abstract

**Fundamento:**

A ressonância magnética cardiovascular (RMC) é o método de referência para avaliação morfofuncional cardíaca. No Brasil, a aplicação de valores de normalidade internacionais enfrenta desafios relacionados à miscigenação populacional, heterogeneidade técnica entre centros e ausência de padronização nacional, o que pode impactar a acurácia diagnóstica.

**Objetivos:**

Comparar valores de normalidade de parâmetros morfofuncionais obtidos por RMC entre uma revisão internacional multicêntrica e uma publicação nacional, discutindo concordância, divergências metodológicas e implicações para a prática clínica brasileira.

**Métodos:**

Revisão analítico-comparativa entre dados sumarizados de uma revisão internacional de valores de referência em RMC e um estudo brasileiro. Foram comparadas médias e desvios-padrão das principais variáveis ventriculares, com avaliação de sobreposição de IC95%, teste t de Welch e tamanho de efeito (Hedges’ g). Propuseram-se pontos de corte baseados em desvios-padrão para gradação de anormalidade.

**Resultados:**

Observou-se concordância global entre as médias na maioria das variáveis. Diferenças estatisticamente significativas ocorreram em poucos parâmetros, principalmente diâmetros ventriculares e massa ventricular esquerda, com tamanhos de efeito pequenos a moderados. As discrepâncias foram atribuídas, em grande parte, a diferenças metodológicas de mensuração e indexação.

**Conclusões:**

Os valores internacionais de normalidade podem ser utilizados na prática clínica brasileira, desde que ajustados por superfície corporal e interpretados à luz de particularidades locais. A padronização de protocolos e estudos multicêntricos nacionais são necessários para maior robustez e validação regional.

## Visual Abstract

**Figure:**
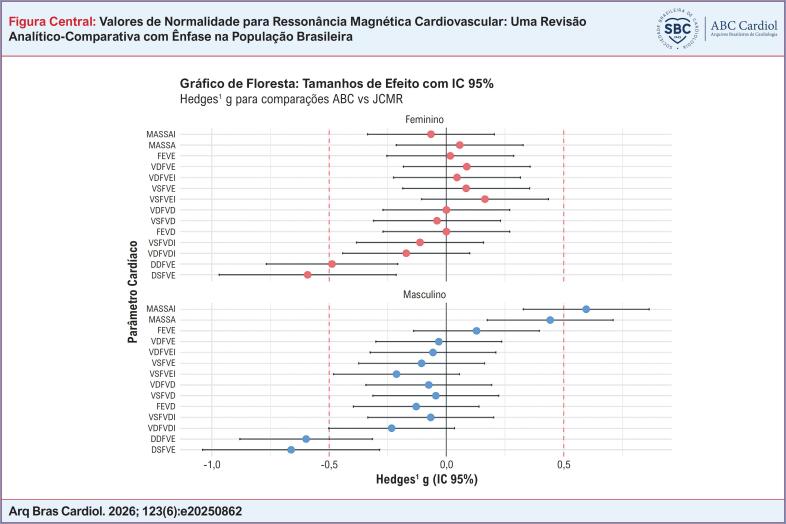
Forest Plot. Efeito da diferença clínica entre as médias e IC.

## Introdução

A ressonância magnética cardíaca (RMC) consolidou-se como método padrão-ouro para avaliação morfofuncional do coração, oferecendo uma combinação única de alta resolução anatômica, caracterização tecidual e análise funcional.^1^ Sua capacidade de fornecer medidas reprodutíveis e tridimensionais de volumes ventriculares, fração de ejeção, massa miocárdica e fluxo sanguíneo a torna indispensável no diagnóstico e acompanhamento de diversas cardiopatias, incluindo cardiomiopatias hipertróficas e arritmogênicas, doenças isquêmicas, cardiopatias congênitas, miocardite, amiloidose, entre outras. Além disso, a RMC apresenta menor variabilidade interobservador em comparação ao ecocardiografia.^2^ É o método mais confiável para avaliação da massa ventricular esquerda, crucial no diagnóstico de hipertrofia.^3,4^ Diferentemente da tomografia e cintilografia, a RMC não emite radiação ionizante e é segura para repetidas avaliações, sendo ideal para acompanhamento de doenças crônicas.^5^

A RMC é uma ferramenta insubstituível na cardiologia moderna, mas sua efetividade no Brasil depende da validação de valores de normalidade locais. A falta de padronização pode levar a subdiagnósticos ou superdiagnósticos, especialmente em populações com características distintas das coortes internacionais. Estudos multicêntricos brasileiros futuros são essenciais para estabelecer diretrizes nacionais confiáveis.

A população brasileira é altamente miscigenada, com influências genéticas europeias, africanas e indígenas, o que pode afetar parâmetros cardíacos. Poucos estudos nacionais definem valores de normalidade para a RMC, e muitos apresentam amostras pequenas ou restritas a regiões específicas. Em 2013, um estudo brasileiro pioneiro^6^ descreveu valores de referência para parâmetros cardíacos obtidos por ressonância magnética em uma população de adultos brasileiros saudáveis. Os autores analisaram medidas como volumes ventriculares, fração de ejeção e massa miocárdica, fornecendo dados relevantes para a prática clínica nacional.

O trabalho destaca particularidades da população brasileira, que apresenta características distintas das coortes internacionais. Seus resultados contribuem para a padronização da interpretação dos exames de RMC no contexto nacional, considerando fatores como diversidade étnica e características regionais. O estudo representa uma base valiosa para futuras pesquisas e para a adequação de protocolos de diagnóstico cardiovascular no Brasil. Ressalta-se, contudo, que não deve ser interpretado como representativo da população brasileira em termos epidemiológicos, mas sim como a melhor evidência nacional disponível para fins comparativos.

Em 2020, uma importante publicação^7^ reuniu valores de normalidade provenientes de múltiplos centros ao redor do mundo e estabeleceu valores de referência normativos para a RMC em adultos e crianças saudáveis. Devido ao seu caráter multicêntrico, internacional e multiétnico, essa publicação passou a ser amplamente utilizada como referência para avaliação clínica e pesquisa em diversos países. Em 2025, houve uma atualização desses dados, com a inclusão de novas coortes, tornando o conjunto ainda mais robusto.^8^ O trabalho fornece parâmetros estratificados por sexo e idade, incluindo volumes ventriculares, fração de ejeção e massa miocárdica. O estudo também destaca a importância de considerar variações populacionais e técnicas na interpretação dos exames. Seus resultados são fundamentais para aprimorar a precisão diagnóstica e a padronização de laudos em RMC, facilitando comparações entre diferentes populações e centros médicos. Ressalta-se que essa revisão internacional utilizou, entre outras fontes, dados da publicação brasileira para compor algumas das médias agrupadas.

A maioria dos serviços de imagem no Brasil ainda se baseia em diferentes referências internacionais, que podem não refletir adequadamente a realidade local. Além disso, os centros utilizam equipamentos de fabricantes distintos, com variações nas sequências de imagem, protocolos e parâmetros de aquisição. A ausência de diretrizes nacionais unificadas dificulta a comparação de resultados entre instituições. A RMC permanece concentrada em grandes centros urbanos, com disponibilidade limitada em regiões periféricas. Considerando a escassez de evidências sobre valores de normalidade no país, esta revisão busca avaliar a possibilidade de utilização dos valores de referência internacionais na prática clínica brasileira.

Diante disso, o presente estudo tem como objetivo comparar os valores de normalidade propostos em uma revisão internacional com os dados obtidos na publicação nacional para discutir as principais divergências metodológicas e clínicas observadas, e propor uma estratégia de padronização com base na realidade brasileira.

## Métodos

Esta revisão segue uma abordagem analítico-comparativa e exploratória, utilizando os valores de normalidade da principal revisão internacional sobre o tema^8^ e os valores do estudo brasileiro.^6^ Foram analisadas as médias e os desvios-padrão (DPs) das principais variáveis, e estabelecidos os graus de dispersão com base nos DPs de ambos os estudos. Avaliou-se o grau de concordância entre os parâmetros e discutiram-se possíveis causas de divergência (fatores étnicos, metodológicos e populacionais). Também foram definidos pontos de corte de normalidade, com ênfase nos diferentes graus de anormalidade (discreto, moderado e importante), utilizando de forma exploratória os valores de 1,5; 3,0; e 4,5 DPs, respectivamente. Vale ressaltar que não foi possível acessar os microdados das coortes, o que inviabilizou análises de distribuição real e ajustes multivariáveis.

### Métodos dos estudos

No estudo brasileiro,^6^ tratou-se de uma publicação original em que os indivíduos realizaram os exames em aparelhos de 1,5 Tesla (Achieva, Philips Medical Systems, Best, The Netherlands; Signa CV/I, GE Medical Systems, Waukesha, WI; e Avanto, Siemens Medical Solutions, Erlangen, Germany). As imagens sincronizadas ao eletrocardiograma foram adquiridas em apneia expiratória (pausa respiratória de 15 segundos), utilizando bobinas cardíacas dedicadas. A aquisição das imagens foi realizada com sequências cine-SSFP (Steady-State Free Precession) para avaliação dos volumes ventriculares e da função sistólica, pelo método de Simpson, com cortes no eixo curto (cobrindo o ventrículo esquerdo da base ao ápice) e imagens adicionais em duas e quatro câmaras.

O pós-processamento e a análise foram realizados por três especialistas experientes utilizando softwares dedicados (ViewForum, da Philips Medical, e Argus, da Siemens Medical Solutions) para a quantificação da fração de ejeção (FEVE e FEVD) e dos volumes ventriculares (VDFVE, VSFVE, VDFVD, VSFVD), além da massa miocárdica do ventrículo esquerdo, com indexação dos valores pela área de superfície corporal (ASC). Para as medições, foram feitas delimitações manuais do endocárdio e do epicárdio em diástole e sístole, excluindo-se o miocárdio trabeculado e os músculos papilares no cálculo da massa ventricular.

A publicação internacional^8^ consiste em uma revisão que realizou uma busca no PubMed para identificar estudos que apresentassem intervalos de referência em RMC. Os critérios gerais e resumidos para inclusão dos dados foram: 1. Tamanho amostral mínimo de 40 indivíduos (ou 40 por sexo), considerado o menor adequado para cálculo de intervalos de referência por métodos paramétricos; 2. Inclusão apenas de coortes saudáveis, excluindo indivíduos com doenças que afetassem os parâmetros analisados (ex.: hipertensão, diabetes). Dados de estudos populacionais (como MESA e UK Biobank) foram filtrados para subgrupos saudáveis; 3. Evitou-se duplicação de coortes em publicações múltiplas.

Foram excluídos estudos que utilizavam técnicas obsoletas de RMC, aqueles com dados faltantes não recuperáveis junto aos autores, descrições metodológicas insuficientes ou inconsistentes, ou métodos não alinhados às diretrizes da *Society for Cardiovascular Magnetic Resonance*. Essa revisão teve como objetivo padronizar diversas variáveis avaliadas na RMC, com abrangência maior do que a publicação brasileira. Assim, apenas os estudos utilizados para análise das variáveis abordadas nesta revisão atual serão mencionados: volume diastólico final do ventrículo esquerdo absoluto e indexado (VDFVE e VDFVEi), volume sistólico final do ventrículo esquerdo absoluto e indexado (VSFVE e VSFVEi), fração de ejeção do ventrículo esquerdo (FEVE), massa do ventrículo esquerdo absoluta e indexada (M e Mi), diâmetros diastólico e sistólico do ventrículo esquerdo (DDVE e DSVE), volume diastólico final do ventrículo direito absoluto e indexado (VDFVD e VDFVDi), volume sistólico final do ventrículo direito absoluto e indexado (VSFVD e VSFVDi) e fração de ejeção do ventrículo direito (FEVD). Vale destacar que a publicação brasileira está entre as incluídas na revisão internacional.^3,6,9-26^

O método para avaliar o ventrículo esquerdo (VE) foi a técnica de cine-SSFP (*Steady-State Free Precession*) em ressonância magnética de 1.5 ou 3T. A maioria dos estudos usa softwares semiautomáticos para análise da função e estrutura do VE e VD, aplicando o método de Simpson em imagens de eixo curto, com inclusão dos músculos papilares na medida do volume cavitário ventricular.

### Análise estatística

A comparação entre os parâmetros dos dois estudos foi conduzida por meio de três abordagens estatísticas complementares. Primeiramente, avaliou-se a sobreposição dos intervalos de confiança (IC95%) das médias, utilizando o erro-padrão (EP) calculado pela fórmula EP = DP / √n. A ausência de sobreposição entre os intervalos de confiança pode ser interpretada como sugestiva de diferença estatisticamente relevante.

Em seguida, aplicou-se o teste t de Welch para amostras independentes, apropriado para situações com variâncias distintas e dados sumarizados, a fim de testar formalmente a hipótese de igualdade entre as médias. Os graus de liberdade foram ajustados segundo a fórmula de Welch, e p-valores bicaudais inferiores a 0,05 foram considerados indicativos de diferença estatisticamente significativa. Para mensurar a relevância prática das diferenças, foi calculado o tamanho de efeito padronizado utilizando o estimador Hedges’ g, que representa uma correção do coeficiente de Cohen's d para amostras de tamanhos diferentes ou reduzidos. O valor do teste "*g" de Hedges* foi interpretado conforme os pontos de corte propostos por Cohen, avaliando seu valor absoluto: diferença desprezível / ausente (< 0,2), pequena diferença (0,2-0,5), moderada diferença (0,5-0,8) ou grande diferença (> 0,8).^27^ As análises estatísticas foram realizadas utilizando a linguagem R (versão 4.4.3).

## Resultados

**Figura 1:**
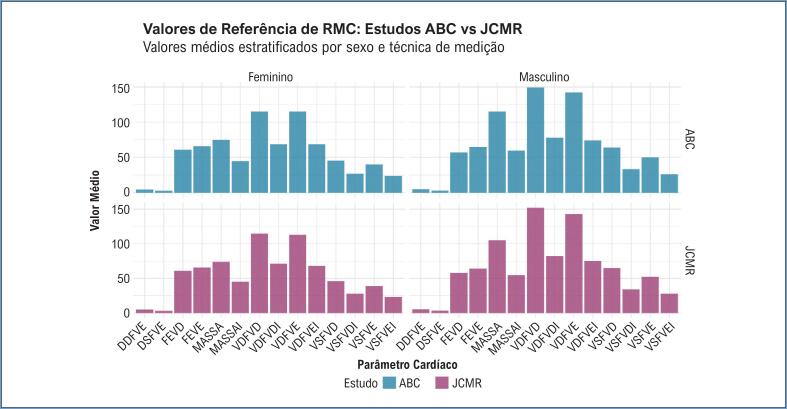
Valores médios das variáveis analisadas.

Foram comparadas as variáveis morfofuncionais cardíacas obtidas por RMC entre os dois estudos. A análise foi estratificada por sexo e realizada sobre os parâmetros descritos na Figura 1.

A inspeção dos intervalos de confiança (IC95%) demonstrou ampla sobreposição na maioria dos parâmetros, sugerindo elevada concordância entre os resultados das amostras. A análise formal pelo teste t de Welch indicou diferenças estatisticamente significativas (p < 0,05) em poucas variáveis. Além da comparação estatística, avaliou-se também a relevância clínica das diferenças por meio do tamanho de efeito padronizado (Hedges’ g), que fornece uma medida interpretável da magnitude da diferença entre grupos.

Entre os homens, observaram-se diferenças significativas na massa absoluta do ventrículo esquerdo (Hedges’ g: 0,44), caracterizando pequena diferença clínica, e na massa indexada (Hedges’ g: 0,59), bem como nos diâmetros ventriculares (DDVE: 0,59 e DSVE: 0,66), estes últimos com efeito clínico moderado. Entre as mulheres, houve diferenças significativas apenas nos diâmetros ventriculares (Hedges’ g: DDVE: 0,48 e DSVE: 0,59), indicando diferenças clínicas discreta e moderada, respectivamente. Para as demais variáveis, as diferenças não foram estatisticamente significativas e apresentaram tamanhos de efeito pequenos (Hedges’ g < 0,5) (Tabela 1 e Figura Central).

**Tabela 1:** Comparação dos valores da revisão internacional com a publicação brasileira

Publicações		Macedo et al (6)					Kawel Bohem et al (8)					p	Hedges’ g	Efeito Clínico
Parâmetros	Sexo	n	m	dp	IC (95%)		n	m	dp	IC (95%)				
FEVE	M	54	64,9	6,1	63,3	66,5	4854	64,0	7,0	63,8	64,1	0,28	0,128	Mínimo
VDFVE	M	54	142,0	26,7	135,0	149,0	4867	143,0	31,0	142,1	143,8	0,78	0,032	Mínimo
VDFVEi	M	54	74,2	13,1	70,7	77,7	4868	75,0	14,0	74,6	75,3	0,65	0,057	Mínimo
VSFVE	M	54	50,1	14,1	46,3	53,9	4857	52,0	18,0	51,4	52,5	0,33	0,105	Mínimo
VSFVEi	M	54	26,3	7,3	24,4	28,2	4847	28,0	8,0	27,7	28,2	0,09	0,212	Pequeno
MASSA	M	54	115,2	26,0	108,0	122,0	4859	105,0	23,0	104,3	105,6	0,005	0,442	Pequeno
MASSAi	M	54	59,8	11,7	56,7	62,9	4850	55,0	8,0	54,7	55,2	0,004	0,596	Médio
DDFVE	M	54	4,9	0,5	4,8	5,0	500	5,2	0,5	5,1	5,2	0,0001	0,598	Médio
DSFVE	M	54	3,2	0,3	3,1	3,3	60	3,4	0,3	3,3	3,4	0,0005	0,662	Médio
FEVD	M	54	57,1	7,1	55,2	59,0	5095	58,0	7,0	57,8	58,1	0,358	0,128	Mínimo
VDFVD	M	54	149,0	33,8	140,0	158,0	5097	152,0	40,0	150,9	153,0	0,52	0,075	Mínimo
VDFVDi	M	54	77,8	16,4	73,4	82,2	5095	82,0	18,0	81,5	82,4	0,066	0,233	Pequeno
VSFVD	M	54	64,1	19,3	59	69,2	5103	65,0	20,0	64,4	65,5	0,73	0,045	Mínimo
VSFVDi	M	54	33,4	9,0	31	35,8	5102	34,0	9,0	33,7	34,2	0,62	0,066	Mínimo
FEVE	F	53	66,1	6,5	64,4	67,8	5209	66,0	6,0	65,8	66,1	0,91	0,016	Mínimo
VDFVE	F	53	115,0	26,0	108,0	122,0	5213	113,0	23,0	112,3	113,6	0,579	0,086	Mínimo
VDFVEi	F	53	68,5	16,8	64,0	73,0	5209	68,0	11,0	67,7	68,2	0,829	0,045	Mínimo
VSFVE	F	53	40,1	17,5	35,4	44,8	5203	39,0	13,0	38,6	39,3	0,65	0,084	Mínimo
VSFVEi	F	53	24,0	11,3	21,0	27,0	5200	23,0	6,0	22,8	23,1	0,52	0,164	Mínimo
MASSA	F	53	74,8	20,1	69,4	80,2	5203	74,0	14,0	73,6	74,3	0,773	0,056	Mínimo
MASSAi	F	53	44,6	11,7	41,5	47,7	5205	45,0	6,0	44,8	45,1	0,80	0,065	Mínimo
DDFVE	F	53	4,6	0,5	4,5	4,7	672	4,8	0,4	4,7	4,8	0,008	0,487	Pequeno
DSFVE	F	53	2,8	0,6	2,6	3,0	60	3,1	0,4	2,9	3,2	0,002	0,591	Médio
FEVD	F	53	61,0	7,8	58,9	63,1	5219	61,0	7,0	60,8	61,1	1,0	0,0	Mínimo
VDFVD	F	53	115,0	26,0	108,0	122,0	5219	115,0	29,0	114,2	115,7	1,0	0,0	Mínimo
VDFVDi	F	53	68,6	15,2	64,5	72,7	5221	71,0	14,0	70,6	71,3	0,25	0,171	Mínimo
VSFVD	F	53	45,4	14,3	41,6	49,2	5216	46,0	15,0	45,5	46,4	0,76	0,040	Mínimo
VSFVDi	F	53	27,1	8,5	24,8	29,4	5214	28,0	8,0	27,7	28,2	0,44	0,112	Mínimo

VDFVE: volume diastólico final do ventrículo esquerdo absoluto; VDFVEi: volume diastólico final do ventrículo esquerdo indexado; VSFVE: volume sistólico final do ventrículo esquerdo absoluto; VSFVEi: volume sistólico final do ventrículo esquerdo indexada; FEVE: fração de ejeção do ventrículo esquerdo; FEVD: fração de ejeção do ventrículo direito; MASSA: massa do ventrículo esquerdo absoluta; MASSi: massa do ventrículo esquerdo indexada; DDVE: diâmetro diastólico do ventrículo esquerdo; DSVE: diâmetro sistólico do ventrículo esquerdo; VDFVD: volume diastólico final do ventrículo direito absoluto; VDFVDi: volume diastólico final do ventrículo direito absoluto indexado; VSFVD: volume sistólico final do ventrículo direito absoluto; VSFVDi: volume sistólico final do ventrículo direito indexada; n: amostra; m: média; DP: desvio padrão; IC: intervalo de confiança; M: mulheres; H: homens. Valor de p significativo < 0,05, teste t de Welch. Efeito clínico das diferença de IC analisado pelo teste "g" de Hedges. Diferença entre as médias é desprezível / ausente (< 0,3), pequena (0,3-0,5), moderada (0,5-0,8) ou grande (> 0,8).

Após constatar a divergência desprezível ou pequena entre as médias das duas publicações, procedeu-se à extração dos valores a partir das médias e dos DPs (1/1,5/2/2,5/3/3,5/4/4,5/5/5,5/6) das principais variáveis de interesse identificadas na revisão internacional, seguida da análise dos pontos de corte com ênfase nos diferentes graus de anormalidade (discreto, moderado e importante). Após avaliação criteriosa, foram utilizados os valores de 1,5; 3; e 4,5 DPs como pontos de corte, respectivamente. Os valores para mulheres encontram-se na Tabela 2 e, para homens, na Tabela 3. Por fim, foi elaborada uma tabela com a proposta de padronização, apresentada nas Tabelas Suplementares S1 e S2.

**Tabela 2:** Médias e desvio padrão para mulheres (8)

						DESVIOS PADRÃO											
VENTRÍCULO ESQUERDO		m	dp	LI	LS		1	1,5	2	2,5	3	3,5	4	4,5	5	5,5	6
FEVE	%	66,0	6,0	57,0	75,0		60,0	57,0	54,0	51,0	48,0	45,0	42,0	39,0	36,0	33,0	30,0
VDFVE	mL	113,0	23,0	78,5	147,5		136,0	147,5	159,0	170,5	182,0	193,5	205,0	216,5	228,0	239,5	251,0
VDFVEi	mL/m^2^	68,0	11,0	51,5	84,5		79,0	84,5	90,0	95,5	101,0	106,5	112,0	117,5	123,0	128,5	134,0
VSFVE	mL	39,0	13,0	19,5	58,5		52,0	58,5	65,0	71,5	78,0	84,5	91,0	97,5	104,0	110,5	117,0
VSFVEi	mL/m^2^	23,0	6,0	14,0	32,0		29,0	32,0	35,0	38,0	41,0	44,0	47,0	50,0	53,0	56,0	59,0
VSVE	mL	74,0	14,0	53,0	95,0		88,0	95,0	102,0	109,0	116,0	123,0	130,0	137,0	144,0	151,0	158,0
VSVEi	mL/m^2^	45,0	6,0	36,0	54,0		51,0	54,0	57,0	60,0	63,0	66,0	69,0	72,0	75,0	78,0	81,0
MASSA	g	74,0	14,0	53,0	95,0		88,0	95,0	102,0	109,0	116,0	123,0	130,0	137,0	144,0	151,0	158,0
MASSAi	g/m^2^	45,0	6,0	36,0	54,0		51,0	54,0	57,0	60,0	63,0	66,0	69,0	72,0	75,0	78,0	81,0
DCVE	L/min	4,9	1,1	3,3	6,6		6,0	6,6	7,1	7,7	8,2	8,8	9,3	9,9	10,4	11,0	11,5
ICVE	L/min/m^2^	3,0	0,6	2,1	3,9		3,6	3,9	4,2	4,5	4,8	5,1	5,4	5,7	6,0	6,3	6,6
Anterosseptal	cm	0,7	0,1	0,5	1,0		0,9	1,0	1,0	1,1	1,2	1,2	1,3	1,4	1,4	1,5	1,6
Inferolateral	cm	0,6	0,1	0,5	0,8		0,8	0,8	0,9	0,9	1,0	1,1	1,1	1,2	1,2	1,3	1,4
DDFVE	cm	4,8	0,4	4,2	5,4		5,2	5,4	5,6	5,8	6,0	6,2	6,4	6,6	6,8	7,0	7,2
DDFVEi	cm/m^2^	3,2	0,4	2,6	3,8		3,6	3,8	4,0	4,2	4,4	4,6	4,8	5,0	5,2	5,4	5,6
DSFVE	cm	3,1	0,4	2,5	3,7		3,5	3,7	3,9	4,1	4,3	4,5	4,7	4,9	5,1	5,3	5,5
DSFVEi	cm/m^2^	1,7	0,4	1,1	2,3		2,1	2,3	2,5	2,7	2,9	3,1	3,3	3,5	3,7	3,9	4,1
**ÁTRIO ESQUERDO**																	
Transversal no 3CH	cm	3,0	1,0	1,5	4,5		4,0	4,5	5,0	5,5	6,0	6,5	7,0	7,5	8,0	8,5	9,0
Indexado	cm/m^2^	2,0	1,0	0,5	3,5		3,0	3,5	4,0	4,5	5,0	5,5	6,0	6,5	7,0	7,5	8,0
Longitudinal no 4CH	cm	5,4	0,7	4,4	6,5		6,1	6,5	6,8	7,2	7,5	7,9	8,2	8,6	8,9	9,3	9,6
Indexado	cm/m^2^	3,4	0,6	2,5	4,3		4,0	4,3	4,6	4,9	5,2	5,5	5,8	6,1	6,4	6,7	7,0
Área no 2CH	cm^2^	18,0	4,0	12,0	24,0		22,0	24,0	26,0	28,0	30,0	32,0	34,0	36,0	38,0	40,0	42,0
Indexado	cm^2^/m^2^	11,0	3,0	6,5	15,5		191,0	182,0	173,0	164,0	155,0	146,0	137,0	128,0	119,0	110,0	101,0
Área no 4Ch	cm^2^	19,0	4,0	13,0	25,0		23,0	25,0	27,0	29,0	31,0	33,0	35,0	37,0	39,0	41,0	43,0
Indexado	cm^2^/m^2^	11,0	2,0	8,0	14,0		13,0	14,0	15,0	16,0	17,0	18,0	19,0	20,0	21,0	22,0	23,0
Volume (Biplanar)	mL	60,0	17,0	34,5	85,5		77,0	85,5	94,0	102,5	111,0	119,5	128,0	136,5	145,0	153,5	162,0
Indexado	mL/m^2^	36,0	9,0	22,5	49,5		45,0	49,5	54,0	58,5	63,0	67,5	72,0	76,5	81,0	85,5	90,0
**OUTROS**																	
Artéria pulmonar	cm	2,2	0,3	1,8	2,5		2,4	2,5	2,7	2,8	2,9	3,0	3,2	3,3	3,4	3,5	3,7
Indexado	cm/m^2^	1,3	0,2	1,1	1,6		1,5	1,6	1,7	1,7	1,8	1,9	2,0	2,1	2,1	2,2	2,3
Raiz da aorta	cm	3,1	0,3	2,6	3,6		3,4	3,6	3,7	3,9	4,1	4,2	4,4	4,6	4,7	4,9	5,1
Indexado	cm/m^2^	1,8	0,2	1,5	2,1		2,0	2,1	2,2	2,3	2,4	2,5	2,6	2,7	2,8	2,9	3,0
Aorta ascendente	cm	3,0	0,6	2,1	3,9		3,6	3,9	4,2	4,5	4,8	5,1	5,4	5,7	6,0	6,3	6,6
Indexado	cm/m^2^	1,8	0,3	1,4	2,3		2,1	2,3	2,4	2,6	2,7	2,9	3,0	3,2	3,3	3,5	3,6
**VENTRÍCULO DIREITO**																	
FEVD	%	61,0	7,0	50,5	71,5		54,0	50,5	47,0	43,5	40,0	36,5	33,0	29,5	26,0	22,5	19,0
VDFVD	mL	115,0	69,0	11,5	218,5		184,0	218,5	253,0	287,5	322,0	356,5	391,0	425,5	460,0	494,5	529,0
VDFVDi	mL/m^2^	71,0	14,0	50,0	92,0		85,0	92,0	99,0	106,0	113,0	120,0	127,0	134,0	141,0	148,0	155,0
VSFVD	mL	46,0	15,0	23,5	68,5		61,0	68,5	76,0	83,5	91,0	98,5	106,0	113,5	121,0	128,5	136,0
VSFVDi	mL/m^2^	28,0	8,0	16,0	40,0		36,0	40,0	44,0	48,0	52,0	56,0	60,0	64,0	68,0	72,0	76,0
VSVD	mL	71,0	20,0	41,0	101,0		91,0	101,0	111,0	121,0	131,0	141,0	151,0	161,0	171,0	181,0	191,0
VSVDi	mL/m^2^	45,0	10,0	30,0	60,0		55,0	60,0	65,0	70,0	75,0	80,0	85,0	90,0	95,0	100,0	105,0
DCVD	L/min	4,6	1,2	2,8	6,4		5,8	6,4	7,0	7,6	8,2	8,8	9,4	10,0	10,6	11,2	11,8
ICVD	L/min/m^2^	2,7	0,7	1,7	3,8		3,4	3,8	4,1	4,5	4,8	5,2	5,5	5,9	6,2	6,6	6,9
**ÁTRIO DIREITO**																	
Transversal no 4CH	cm	4,1	0,7	3,1	5,2		4,8	5,2	5,5	5,9	6,2	6,6	6,9	7,3	7,6	8,0	8,3
Indexado	cm/m^2^	2,6	0,4	2,0	3,2		3,0	3,2	3,4	3,6	3,8	4,0	4,2	4,4	4,6	4,8	5,0
Longitudinal no 4CH	cm	4,9	0,7	3,9	6,0		5,6	6,0	6,3	6,7	7,0	7,4	7,7	8,1	8,4	8,8	9,1
Indexado	cm/m^2^	3,1	0,5	2,4	3,9		3,6	31,6	30,1	28,6	27,1	25,6	24,1	22,6	21,1	19,6	18,1
Área no 4Ch	cm^2^	18,0	0,4	17,4	18,6		18,4	18,6	18,8	19,0	19,2	19,4	19,6	19,8	20,0	20,2	20,4
Indexado	cm^2^/m^2^	11,0	0,2	10,7	11,3		11,2	11,3	11,4	11,5	11,6	11,7	11,8	11,9	12,0	12,1	12,2
Volume (Biplanar)	mL	53,0	14,0	32,0	74,0		23,0	74,0	81,0	88,0	95,0	102,0	109,0	116,0	123,0	130,0	137,0
Indexado	mL/m^2^	35,0	10,0	20,0	50,0		38,2	50,0	55,0	60,0	65,0	70,0	75,0	80,0	85,0	90,0	95,0

VDFVE: volume diastólico final do ventrículo esquerdo absoluto; VDFVEi: volume diastólico final do ventrículo esquerdo indexado; VSFVE: volume sistólico final do ventrículo esquerdo absoluto; VSFVEi: volume sistólico final do ventrículo esquerdo indexada; FEVE: fração de ejeção do ventrículo esquerdo; FEVD: fração de ejeção do ventrículo direito; MASSA: massa do ventrículo esquerdo absoluta; MASSi: massa do ventrículo esquerdo indexada; DDVE: diâmetro diastólico do ventrículo esquerdo; DSVE: diâmetro sistólico do ventrículo esquerdo; VDFVD: volume diastólico final do ventrículo direito absoluto; VDFVDi: volume diastólico final do ventrículo direito absoluto indexado; VSFVD: volume sistólico final do ventrículo direito absoluto; VSFVDi: volume sistólico final do ventrículo direito indexada; m: média; DP: desvio padrão; quatro câmaras (4C), três câmaras (3C).

**Tabela 3:** Médias e desvio padrão para homens (8)

							DESVIOS PADRÃO										
VENTRÍCULO ESQUERDO		m	dp	LI	LS		1	1,5	2	2,5	3	3,5	4	4,5	5	5,5	6
FEVE	%	64,0	7,0	53,5	74,5	57,0	53,5	50,0	46,5	43,0	39,5	36,0	32,5	29,0	25,5	22,0	64,0
VDFVE	mL	143,0	31,0	96,5	189,5	174,0	189,5	205,0	220,5	236,0	251,5	267,0	282,5	298,0	313,5	329,0	143,0
VDFVEi	mL/m^2^	75,0	14,0	54,0	96,0	89,0	96,0	103,0	110,0	117,0	124,0	131,0	138,0	145,0	152,0	159,0	75,0
VSFVE	mL	52,0	18,0	25,0	79,0	70,0	79,0	88,0	97,0	106,0	115,0	124,0	133,0	142,0	151,0	160,0	52,0
VSFVEi	mL/m^2^	28,0	8,0	16,0	40,0	36,0	40,0	44,0	48,0	52,0	56,0	60,0	64,0	68,0	72,0	76,0	28,0
VSVE	mL	91,0	19,0	62,5	119,5	110,0	119,5	129,0	138,5	148,0	157,5	167,0	176,5	186,0	195,5	205,0	91,0
VSVEi	mL/m^2^	47,0	9,0	33,5	60,5	56,0	60,5	65,0	69,5	74,0	78,5	83,0	87,5	92,0	96,5	101,0	47,0
MASSA	g	105,0	23,0	70,5	139,5	128,0	139,5	151,0	162,5	174,0	185,5	197,0	208,5	220,0	231,5	243,0	105,0
MASSAi	g/m^2^	55,0	8,0	43,0	67,0	63,0	67,0	71,0	75,0	79,0	83,0	87,0	91,0	95,0	99,0	103,0	55,0
DCVE	L/min	6,1	1,3	4,2	8,1	7,4	8,1	8,7	9,4	10,0	10,7	11,3	12,0	12,6	13,3	13,9	6,1
ICVE	L/min/m^2^	3,2	0,6	2,3	4,1	3,8	4,1	4,4	4,7	5,0	5,3	5,6	5,9	6,2	6,5	6,8	3,2
Anterosseptal	cm	0,9	0,2	0,7	1,1	1,1	1,1	1,2	1,3	1,4	1,5	1,5	1,6	1,7	1,8	1,9	0,9
Inferolateral	cm	0,8	0,1	0,6	1,0	0,9	1,0	1,1	1,1	1,2	1,3	1,3	1,4	1,5	1,6	1,6	0,8
DDFVE	cm	5,2	0,5	4,5	6,0	5,7	6,0	6,2	6,5	6,7	7,0	7,2	7,5	7,7	8,0	8,2	5,2
DDFVEi	cm/m^2^	2,6	0,3	2,2	3,1	2,9	3,1	3,2	3,4	3,5	3,7	3,8	4,0	4,1	4,3	4,4	2,6
DSFVE	cm	3,4	0,3	3,0	3,9	3,7	3,9	4,0	4,2	4,3	4,5	4,6	4,8	4,9	5,1	5,2	3,4
DSFVEi	cm/m^2^	1,7	0,2	1,4	2,0	1,9	2,0	2,1	2,2	2,3	2,4	2,5	2,6	2,7	2,8	2,9	1,7
**ÁTRIO ESQUERDO**																	
Transversal no 3CH	cm	3,0	0,5	2,3	3,8	3,5	3,8	4,0	4,3	4,5	4,8	5,0	5,3	5,5	5,8	6,0	3,0
Indexado	cm/m^2^	1,8	0,5	1,1	2,6	2,3	2,6	2,8	3,1	3,3	3,6	3,8	4,1	4,3	4,6	4,8	1,8
Longitudinal no 4CH	cm	5,7	0,7	4,7	6,8	6,4	6,8	7,1	7,5	7,8	8,2	8,5	8,9	9,2	9,6	9,9	5,7
Indexado	cm/m^2^	3,1	0,5	2,4	3,9	3,6	3,9	4,1	4,4	4,6	4,9	5,1	5,4	5,6	5,9	6,1	3,1
Área no 2CH	cm^2^	21,0	4,0	15,0	27,0	25,0	27,0	29,0	31,0	33,0	35,0	37,0	39,0	41,0	43,0	45,0	21,0
Indexado	cm^2^/m^2^	11,0	2,0	8,0	14,0	13,0	14,0	15,0	16,0	17,0	18,0	19,0	20,0	21,0	22,0	23,0	11,0
Área no 4Ch	cm^2^	21,0	4,0	15,0	27,0	25,0	27,0	29,0	31,0	33,0	35,0	37,0	39,0	41,0	43,0	45,0	21,0
Indexado	cm^2^/m^2^	11,0	2,0	8,0	14,0	13,0	14,0	15,0	16,0	17,0	18,0	19,0	20,0	21,0	22,0	23,0	11,0
Volume (Biplanar)	mL	68,0	22,0	35,0	101,0	90,0	101,0	112,0	123,0	134,0	145,0	156,0	167,0	178,0	189,0	200,0	68,0
Indexado	mL/m^2^	36,0	10,0	21,0	51,0	46,0	51,0	56,0	61,0	66,0	71,0	76,0	81,0	86,0	91,0	96,0	36,0
**OUTROS**																	
Artéria pulmonar	cm	2,3	0,3	1,9	2,7	2,6	2,7	2,8	3,0	3,1	3,2	3,4	3,5	3,6	3,7	3,9	2,3
Indexado	cm/m^2^	1,2	0,1	1,0	1,5	1,4	1,5	1,5	1,6	1,7	1,7	1,8	1,9	1,9	2,0	2,1	1,2
Raiz da aorta	cm	3,5	0,4	2,9	4,1	3,9	4,1	4,3	4,5	4,7	4,9	5,1	5,3	5,5	5,7	5,9	3,5
Indexado	cm/m^2^	1,8	0,2	1,5	2,1	2,0	2,1	2,2	2,3	2,4	2,5	2,6	2,7	2,8	2,9	3,0	1,8
Aorta ascendente	cm	3,3	0,5	2,6	4,1	3,8	4,1	4,3	4,6	4,8	5,1	5,3	5,6	5,8	6,1	6,3	3,3
Indexado	cm/m^2^	1,7	0,3	1,3	2,2	2,0	2,2	2,3	2,5	2,6	2,8	2,9	3,1	3,2	3,4	3,5	1,7
**VENTRÍCULO DIREITO**																	
FEVD	%	58,0	7,0	47,5	68,5	51,0	47,5	44,0	40,5	37,0	33,5	30,0	26,5	23,0	19,5	16,0	58,0
VDFVD	mL	152,0	40,0	92,0	212,0	192,0	212,0	232,0	252,0	272,0	292,0	312,0	332,0	352,0	372,0	392,0	152,0
VDFVDi	mL/m^2^	82,0	18,0	55,0	109,0	100,0	109,0	118,0	127,0	136,0	145,0	154,0	163,0	172,0	181,0	190,0	82,0
VSFVD	mL	65,0	20,0	35,0	95,0	85,0	95,0	105,0	115,0	125,0	135,0	145,0	155,0	165,0	175,0	185,0	65,0
VSFVDi	mL/m^2^	34,0	9,0	20,5	47,5	43,0	47,5	52,0	56,5	61,0	65,5	70,0	74,5	79,0	83,5	88,0	34,0
VSVD	mL	89,0	29,0	45,5	132,5	118,0	132,5	147,0	161,5	176,0	190,5	205,0	219,5	234,0	248,5	263,0	89,0
VSVDi	ml/m^2^	48,0	14,0	27,0	69,0	62,0	69,0	76,0	83,0	90,0	97,0	104,0	111,0	118,0	125,0	132,0	48,0
DCVD	L/min	5,5	1,6	3,1	7,9	7,1	7,9	8,7	9,5	10,3	11,1	11,9	12,7	13,5	14,3	15,1	5,5
ICVD	L/min/m^2^	2,9	0,9	1,6	4,3	3,8	4,3	4,7	5,2	5,6	6,1	6,5	7,0	7,4	7,9	8,3	2,9
**ÁTRIO DIREITO**																	
Transversal no 4CH	cm	4,6	0,6	3,7	5,5	5,2	5,5	5,8	6,1	6,4	6,7	7,0	7,3	7,6	7,9	8,2	4,6
Indexado	cm/m^2^	2,5	0,4	1,9	3,1	2,9	3,1	3,3	3,5	3,7	3,9	4,1	4,3	4,5	4,7	4,9	2,5
Longitudinal no 4CH	cm	5,2	0,7	4,2	6,3	5,9	6,3	6,6	7,0	7,3	7,7	8,0	8,4	8,7	9,1	9,4	5,2
Indexado	cm/m^2^	2,8	0,5	2,1	3,6	3,3	29,6	27,8	26,1	24,3	22,6	20,8	19,1	17,3	15,6	13,8	2,8
Área no 4Ch	cm^2^	21,0	5,0	13,5	28,5	26,0	28,5	31,0	33,5	36,0	38,5	41,0	43,5	46,0	48,5	51,0	21,0
Indexado	cm^2^/m^2^	11,0	3,0	6,5	15,5	14,0	15,5	17,0	18,5	20,0	21,5	23,0	24,5	26,0	27,5	29,0	11,0
Volume (Biplanar)	ml	65,0	20,0	35,0	95,0	182,0	95,0	105,0	115,0	125,0	135,0	145,0	155,0	165,0	175,0	185,0	65,0
Indexado	ml/m^2^	38,0	12,0	20,0	56,0	533,0	56,0	62,0	68,0	74,0	80,0	86,0	92,0	98,0	104,0	110,0	38,0

VDFVE: volume diastólico final do ventrículo esquerdo absoluto; VDFVEi: volume diastólico final do ventrículo esquerdo indexado; VSFVE: volume sistólico final do ventrículo esquerdo absoluto; VSFVEi: volume sistólico final do ventrículo esquerdo indexada; FEVE: fração de ejeção do ventrículo esquerdo; FEVD: fração de ejeção do ventrículo direito; MASSA: massa do ventrículo esquerdo absoluta; MASSi: massa do ventrículo esquerdo indexada; DDVE: diâmetro diastólico do ventrículo esquerdo; DSVE: diâmetro sistólico do ventrículo esquerdo; VDFVD: volume diastólico final do ventrículo direito absoluto; VDFVDi: volume diastólico final do ventrículo direito absoluto indexado; VSFVD: volume sistólico final do ventrículo direito absoluto; VSFVDi: volume sistólico final do ventrículo direito indexada; m: média; DP: desvio padrão; quatro câmaras (4C), três câmaras (3C).

## Discussão

A utilização dos valores de referência estabelecidos pela revisão internacional^8^ na prática clínica brasileira merece uma análise criteriosa. Foi demonstrada nesta publicação, uma concordância global entre médias populacionais dos parâmetros da revisão internacionais e os valores obtidos no estudo brasileiro,^6^ abrindo caminho para a padronização dos exames de RMC no país. Esta abordagem traria significativas vantagens, incluindo a possibilidade de adoção imediata de parâmetros validados internacionalmente, a harmonização com centros de referência globais e a facilitação de estudos multicêntricos.

Destacamos que a análise da diferença estatística pelo teste de Welch foi realizada para garantir a integridade cientifica da publicação, mas para a avaliação do efeito clínico, foi utilizado o teste "*g de Hedges"*, que auxilia a interpretar se a diferença entre grupos é apenas estatisticamente significativa ou se tem uma relevância prática, determinando ainda o tamanho do efeito clínico. O teste indica se a diferença entre as médias é desprezível / ausente (< 0,2), pequena (< 0,5), moderada (0,5-0,8) ou grande (> 0,8).

Ademais, nos casos em que foi observada uma divergência moderada entre os conjuntos de dados: massa indexada, DDVE e DSVE, em homens; DSFVE, em mulheres, ressalta-se que para as medidas dos diâmetros diastólico e sistólico do ventrículo esquerdo foram empregadas diferentes metodologias nas duas publicações, sendo que no estudo brasileiro as médias eram adquiridas no longitudinal quatro câmaras, já em desuso, enquanto na revisão internacional era feito no eixo curto. Em relação à variável massa indexada, pode haver alguma variabilidade nesta medida em virtude da complexa morfologia na transição AE e VE, dificultando a delimitação do miocárdio do VE e da parede atrial. Em relação à variável VDFVDi, que apresentou discreta diferença clínica, sem diferença estatística, em virtude da complexa morfologia e complacência do ventrículo direito, parece haver variabilidade nesta medida. Valores normativos do volume do VD podem diferir até 20-30% devido a variações metodológicas e características da amostra populacional.^3^

Para categorizar a dispersão dos volumes dos ventrículos do coração em uma amostra normal (distribuição gaussiana), a metodologia mais comumente utilizada e estatisticamente robusta é baseada nos DPs da média, que permite uma classificação objetiva das alterações (discreta, moderada, importante). Deve ser ressaltado, que pela ausência de padronização formal para esta específica classificação, foi realizada uma avaliação exploratória seguida de possível sugestão. Dentre as opções que foram consideradas, podemos citar:

Primeiro – 1, 2, 3 DP: tem como vantagem ser simples e alinhada com a regra empírica (68-95-99,7% da população dentro de 1, 2, 3 DP); tem como limitação o fato de ser muito ampla para alterações discretas (1 DP já cobre ∼16% dos extremos); segunda opção – 1.5, 3, 4.5 DP - tem como vantagem ampliar a faixa de "normal" (1.5 DP cobre ∼86% da população), reduzindo falsos positivos para alterações discretas e como principal limitação que os cortes muito espaçados podem subestimar gradações; terceira opção – 2, 3, 4 DP – que teria como vantagem ser mais rigorosa para definir a alteração "discreta" (2 DP = ∼5% dos extremos) e útil para detectar alterações clinicamente relevantes. Como limitação, deve ser citado que pode ser muito restritiva se a variabilidade normal for alta.

Após meticulosa consideração foi definido sugerir pela segunda opção - 1.5, 3, 4.5 DP - como o principal objetivo de evitar superdiagnósticos de alterações discretas (1.5 DP é menos sensível que 1 DP) e priorizar especificidade (ex.: só classificar como "moderada" acima de 3 DP, cobrando maior desvio da média). Portanto, a definição de 1.5 DP para alteração "discreta" equilibra sensibilidade/especificidade, 3 DP para alteração "moderada" sinaliza desvios claramente anormais e 4.5 DP para "importante" capta casos raros e graves.^3^ A exceção foi a classificação da FEVE para homens e mulheres, que teve pequeno ajuste para se adaptar melhor os valores já consagrados na literatura e prática clínica. Reforça-se que os pontos de corte não constam nas recomendações da SCMR e não devem ser interpretados como *thresholds* validados.

O trabalho de Petersen et al.^16^ demonstrou que valores de referência obtidos em populações caucasianas mostraram boa concordância com outras populações quando adequadamente ajustados pela ASC. Esses achados sugerem que, com os devidos cuidados metodológicos, a aplicação de valores internacionais em nossa população pode ser clinicamente válida.

Independentemente do grau de concordância encontrado, os resultados desta análise podem contribuir para o debate sobre a padronização da RMC no Brasil. As implicações práticas de uma eventual adoção desses valores de referência são amplas. Do ponto de vista clínico, permitiria maior segurança na interpretação dos exames e melhor comparabilidade entre instituições. No âmbito da gestão em saúde, representaria economia de recursos ao evitar a necessidade de estudos locais extensivos para estabelecimento de parâmetros próprios. Para a pesquisa científica, facilitaria a integração com estudos internacionais e a participação em consórcios globais.

Além disso, os resultados poderão informar as sociedades científicas brasileiras na elaboração de diretrizes nacionais, ajudando a equilibrar a adoção de padrões internacionais com as particularidades de nossa população.

### Limitações

Este estudo apresenta algumas limitações que devem ser consideradas na interpretação dos resultados. O estudo brasileiro apresenta provável viés de seleção, com uma amostra menor e regionalizada, composta predominantemente por indivíduos avaliados em centros do Sudeste. Essa restrição levanta dúvidas sobre a validade desses parâmetros para outras regiões do país, especialmente Norte e Nordeste, onde características populacionais e ambientais podem diferir significativamente. Outro ponto relevante é a variação do índice de massa corporal (IMC) na população brasileira conforme a região, com prevalência de obesidade variando de 9,9% (Nordeste) a 15,9% (Sul),^28^ ilustrando a importância de futuros estudos nacionais mais abrangentes. A revisão internacional, por sua vez, utiliza ajustes e estratificações que não estão disponíveis no estudo brasileiro.

Este estudo não tem como objetivo validar formalmente os valores internacionais para a população brasileira. Trata-se de uma análise de concordância global entre médias sumarizadas, sem acesso a microdados nem ajustes individuais por idade, ASC ou etnia. Por esse motivo, não foram realizados ajustes por fatores demográficos (idade, altura, BSA, etnia). A comparação entre as coortes não considera que distribuições etárias, composição étnica e BSA podem diferir substancialmente entre os estudos. A miscigenação característica da população brasileira contrasta com coortes internacionais predominantemente europeias ou norte-americanas, o que pode afetar a extrapolação dos resultados. Tais limitações reforçam a necessidade de cautela na interpretação e aplicação dos achados.

Não foi possível acessar os microdados das coortes; sem esses dados individuais, a comparação depende de pressupostos fortes (como normalidade e simetria) e impede análises da distribuição real e ajustes multivariáveis, o que pode reduzir a confiabilidade dos testes e do cálculo dos intervalos de referência. A impossibilidade de ajustar por idade, BSA, etnia, estratificações adicionais ou explorar a distribuição real limita conclusões mais robustas sobre equivalência populacional.

Quanto à variabilidade técnica, a natureza multicêntrica das publicações implica potenciais impactos na análise e comparação. Os dois trabalhos apresentam diferenças metodológicas relevantes — equipamentos, protocolos, planos de mensuração, softwares e critérios de inclusão/exclusão de músculos papilares. Ambos utilizam cine-SSFP como técnica base para avaliação morfofuncional, aplicam o método de Simpson para quantificação volumétrica e seguem recomendações alinhadas às diretrizes da SCMR para aquisição e análise de volumes e função ventricular. No entanto, reconhecemos que tais diferenças limitam comparações diretas e qualquer inferência de equivalência populacional. Por isso, deve-se enfatizar a natureza analítico-comparativa exploratória do estudo, sujeita a vieses metodológicos, não configurando validação formal entre coortes.

Além disso, a realidade operacional dos serviços de imagem no Brasil apresenta heterogeneidade considerável em termos de equipamentos e protocolos, fator que pode comprometer a uniformidade dos resultados.

### Perspectivas Futuras

Recomenda-se que a adoção dos valores da revisão internacional na prática clínica brasileira seja acompanhada de iniciativas complementares. Seria desejável a realização de estudos futuros com amplas coortes brasileiras, representativas das diferentes regiões do país e dos setores público e privado, com atenção especial a subgrupos populacionais específicos, como faixas etárias distintas, atletas, indivíduos com obesidade ou grupos étnicos particulares. Idealmente, tais estudos deveriam disponibilizar microdados, permitir modelos ajustados e possibilitar reamostragem ponderada. Da mesma forma, a padronização dos protocolos de aquisição e análise dos exames de RMC entre os centros brasileiros, seguindo as diretrizes propostas, é fundamental para garantir a confiabilidade e a comparabilidade dos resultados.

## Conclusões

Neste contexto, parece razoável afirmar que há concordância global entre as médias populacionais, com evidências suficientes para sugerir que os valores de referência da revisão internacional podem ser aplicados à população brasileira, desde que observados os devidos cuidados. Essa estratégia configura uma alternativa viável e potencialmente benéfica, desde que acompanhada de avaliações criteriosas das particularidades locais, das faixas etárias correspondentes e das metodologias de exame utilizadas. Tal abordagem pode representar um avanço importante para a imagem cardiovascular no país, permitindo maior padronização dos exames e melhor integração com a comunidade científica internacional, sem desconsiderar as características específicas da população brasileira.

Entretanto, ressalta-se a importância de interpretar os resultados com cautela ao extrapolá-los para a prática clínica, especialmente nas áreas limítrofes entre normalidade e patologia. Além disso, os pontos de corte propostos (1,5 / 3,0 / 4,5 DP) devem ser entendidos como uma sugestão pragmática e exploratória voltada à aplicação clínica, e não como recomendações normativas.

## Data Availability

Os conteúdos subjacentes ao texto da pesquisa estão contidos no manuscrito.

## *Material suplementar

Material suplementar Para informação adicional, por favor, clique aqui.
