# Supplementary material for: Valores de Normalidade para Ressonância Magnética Cardiovascular: Uma Revisão Analítico-Comparativa com Ênfase na População Brasileira
Source: Arq Bras Cardiol. 2026 Jul 21;123(6):e20250862. [Article in Portuguese] doi: 10.36660/abc.20250862 (PMC13399083; doi:10.36660/abc.20250862)
Supplement: Material suplementar [file 0066-782X-abc-123-6-e20250862-Suppl01.pdf]

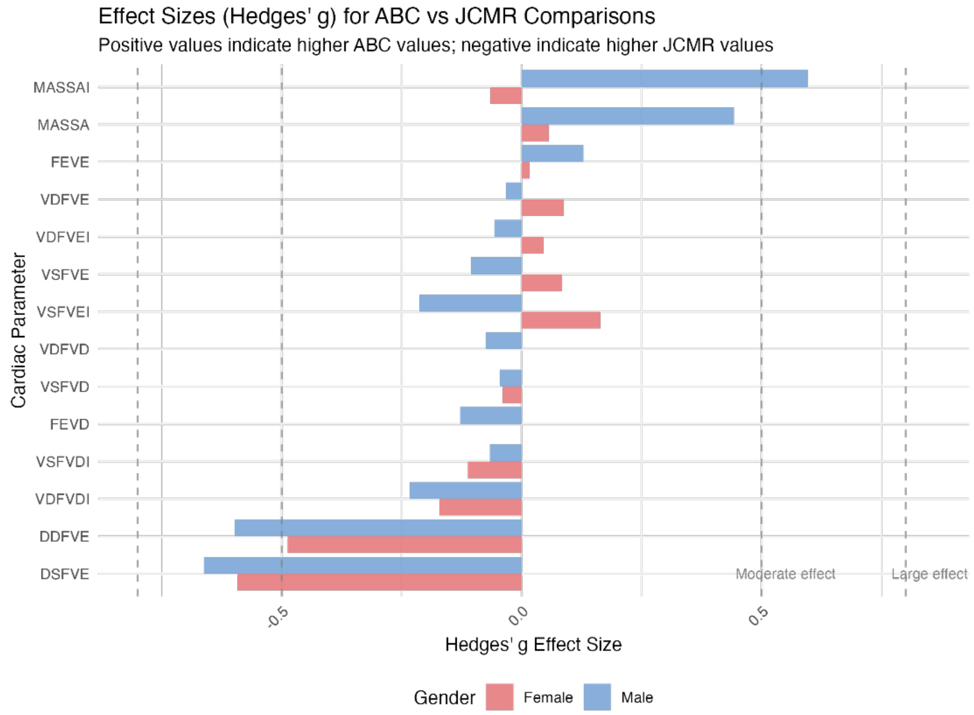

Figura 1 Suplemento. Efeito da diferença clínica entre as médias e IC.

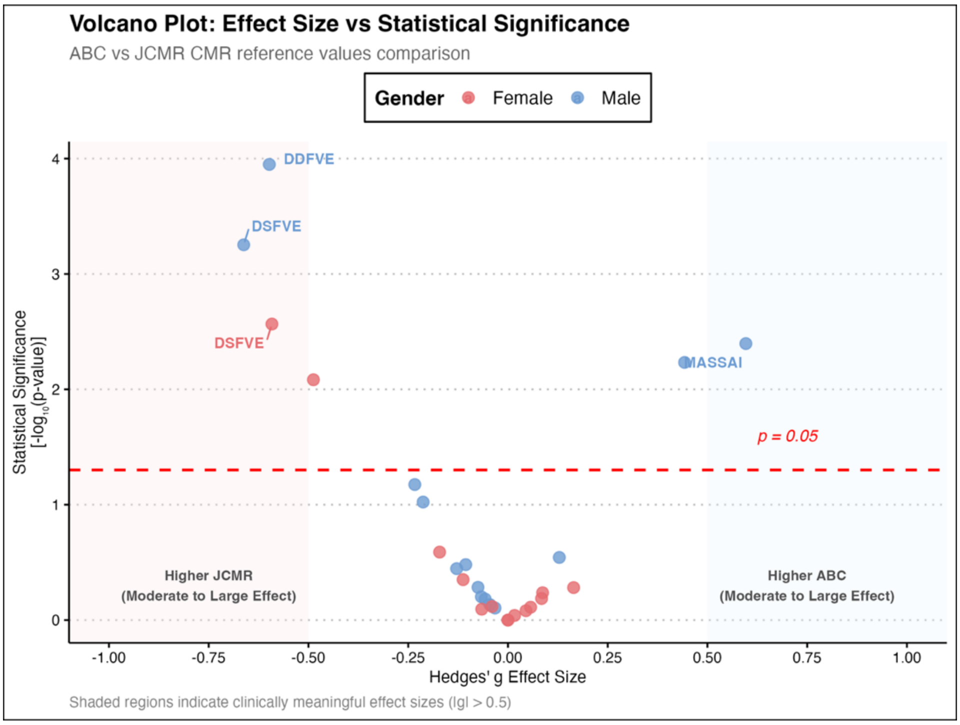

Figura 2 Suplemento. Volcano Plot. Tamanho do efeito e significância estatística.

Tabela 1 Suplemento. Valores normais propostos para mulheres									
**VENTRÍCULO ESQUERDO**		**NORMAL**		**DISCRETO**		**MODERADO**		**IMPORTANTE**	
**Fração de ejeção**	%	54,0	75,0	54,0	45,0	45,0	36,0	<	36,0
**Volume diastólico final**	ml	78,5	147,5	147,5	182,0	182,0	216,5	>	216,5
**Indexado**	ml/m2	51,5	84,5	84,5	101,0	101,0	117,5	>	117,5
**Volume sistólico final**	ml	19,5	58,5	58,5	78,0	78,0	97,5	>	97,5
**Indexado**	ml/m2	14,0	32,0	32,0	41,0	41,0	50,0	>	50,0
**Volume sistólico**	ml	53,0	95,0	95,0	116,0	116,0	137,0	>	137,0
**Indexado**	ml/m2	36,0	54,0	54,0	63,0	63,0	72,0	>	72,0
**Massa ventricular**	g	53,0	95,0	95,0	116,0	116,0	137,0	>	137,0
**Indexado**	g/m2	36,0	54,0	54,0	63,0	63,0	72,0	>	72,0
**Débito cardíaco**	L/min	3,3	6,6	6,6	8,2	8,2	9,9	>	9,9
**Índice cardíaco**	L/min/m2	2,1	3,9	3,9	4,8	4,8	5,7	>	5,7
**Diâmetro do septo**	cm	0,5	1,0	1,0	1,2	1,2	1,4	>	1,4
**Diâmetro inferolateral**	cm	0,5	0,8	0,8	1,0	1,0	1,2	>	1,2
**Diâmetro diastólico**	cm	4,2	5,4	5,4	6,0	6,0	6,6	>	6,6
**Indexado**	cm/m2	2,6	3,8	3,8	4,4	4,4	5,0	>	5,0
**Diâmetro sistólico**	cm	2,5	3,7	3,7	4,3	4,3	4,9	>	4,9
**Indexado**	cm/m2	1,1	2,3	2,3	2,9	2,9	3,5	>	3,5
**ÁTRIO ESQUERDO**		**NORMAL**		**DISCRETO**		**MODERADO**		**IMPORTANTE**	
**Diâmetro transversal 3C**	cm	1,5	4,5	4,5	6,0	6,0	7,5	>	7,5
**Indexado**	cm/m2	0,5	3,5	3,5	5,0	5,0	6,5	>	6,5
**Diâmetro longitudinal 4C**	cm	4,4	6,5	6,5	7,5	7,5	8,6	>	8,6
**Indexado**	cm/m2	2,5	4,3	4,3	5,2	5,2	6,1	>	6,1
**Área 2C**	cm2	12,0	24,0	24,0	30,0	30,0	36,0	>	36,0
**Indexado**	cm2/m2	6,5	15,5	182,0	155,0	155,0	128,0	>	128,0
**Área 4C**	cm2	13,0	25,0	25,0	31,0	31,0	37,0	>	37,0
**Indexado**	cm2/m2	8,0	14,0	14,0	17,0	17,0	20,0	>	20,0
**Volume (Biplanar)**	ml	34,5	85,5	85,5	111,0	111,0	136,5	>	136,5
**Indexado**	ml/m2	22,5	49,5	49,5	63,0	63,0	76,5	>	76,5
**OUTROS**		**NORMAL**		**DISCRETO**		**MODERADO**		**IMPORTANTE**	

**Diâmetro da artéria pulmonar**	cm	1,8	2,5	2,5	2,9	2,9	3,3	>	3,3
**Indexado**	cm/m2	1,1	1,6	1,6	1,8	1,8	2,1	>	2,1
**Diâmetro da raiz da aorta**	cm	2,6	3,6	3,6	4,1	4,1	4,6	>	4,6
**Indexado**	cm/m2	1,5	2,1	2,1	2,4	2,4	2,7	>	2,7
**Diâmetro aorta ascendente**	cm	2,1	3,9	3,9	4,8	4,8	5,7	>	5,7
**Indexado**	cm/m2	1,4	2,3	2,3	2,7	2,7	3,2	>	3,2
**VENTRÍCULO DIREITO**		**NORMAL**		**DISCRETO**		**MODERADO**		**IMPORTANTE**	
**Fração de ejeção**	%	47,0	71,5	47,0	36,5	36,5	26,0	>	26,0
**Volume diastólico final**	ml	11,5	218,5	218,5	322,0	322,0	425,5	>	425,5
**Indexado**	ml/m2	50,0	92,0	92,0	113,0	113,0	134,0	>	134,0
**Volume sistólico final**	ml	23,5	68,5	68,5	91,0	91,0	113,5	>	113,5
**Indexado**	ml/m2	16,0	40,0	40,0	52,0	52,0	64,0	>	64,0
**Volume sistólico**	ml	41,0	101,0	101,0	131,0	131,0	161,0	>	161,0
**Indexado**	ml/m2	30,0	60,0	60,0	75,0	75,0	90,0	>	90,0
**Débito cardíaco**	L/min	2,8	6,4	6,4	8,2	8,2	10,0	>	10,0
**Índice cardíaco**	L/min/m2	1,7	3,8	3,8	4,8	4,8	5,9	>	5,9
**ÁTRIO DIREITO**		**NORMAL**		**DISCRETO**		**MODERADO**		**IMPORTANTE**	
**Diâmetro transversal 4C**	cm	3,1	5,2	5,2	6,2	6,2	7,3	>	7,3
**Indexado**	cm/m2	2,0	3,2	3,2	3,8	3,8	4,4	>	4,4
**Diâmetro longitudinal 4C**	cm	3,9	6,0	6,0	7,0	7,0	8,1	>	8,1
**Indexado**	cm/m2	2,4	3,9	3,9	4,6	4,6	5,4	>	5,4
**Área 4C**	cm2	17,4	18,6	18,6	19,2	19,2	19,8	>	19,8
**Indexado**	cm2/m2	10,7	11,3	11,3	11,6	11,6	11,9	>	11,9
**Volume (Biplanar)**	ml	32,0	74,0	74,0	95,0	95,0	116,0	>	116,0
**Indexado**	ml/m2	20,0	50,0	50,0	65,0	65,0	80,0	>	80,0

Valores normais propostos para mulheres.

Tabela 2 Suplemento. Valores normais propostos para homens									
**VENTRÍCULO ESQUERDO**		**NORMAL**		**DISCRETO**		**MODERADO**		**IMPORTANTE**	
**Fração de ejeção**	%	54,0	74,5	54,0	39,5	39,5	29,0	<	29,0
**Volume diastólico final**	ml	96,5	189,5	189,5	236,0	236,0	282,5	>	282,5
**Indexado**	ml/m2	54,0	96,0	96,0	117,0	117,0	138,0	>	138,0
**Volume sistólico final**	ml	25,0	79,0	79,0	106,0	106,0	133,0	>	133,0
**Indexado**	ml/m2	16,0	40,0	40,0	52,0	52,0	64,0	>	64,0
**Volume sistólico**	ml	62,5	119,5	119,5	148,0	148,0	176,5	>	176,5
**Indexado**	ml/m2	33,5	60,5	60,5	74,0	74,0	87,5	>	87,5
**Massa ventricular**	g	70,5	139,5	139,5	174,0	174,0	208,5	>	208,5
**Indexado**	g/m2	43,0	67,0	67,0	79,0	79,0	91,0	>	91,0
**Débito cardíaco**	L/min	4,2	8,1	8,1	10,0	10,0	12,0	>	12,0
**Índice cardíaco**	L/min/m2	2,3	4,1	4,1	5,0	5,0	5,9	>	5,9
**Diâmetro do septo**	cm	0,7	1,1	1,1	1,4	1,4	1,6	>	1,6
**Diâmetro inferolateral**	cm	0,6	1,0	1,0	1,2	1,2	1,4	>	1,4
**Diâmetro diastólico**	cm	4,5	6,0	6,0	6,7	6,7	7,5	>	7,5
**Indexado**	cm/m2	2,2	3,1	3,1	3,5	3,5	4,0		4,0
**Diâmetro sistólico**	cm	3,0	3,9	3,9	4,3	4,3	4,8	>	4,8
**Indexado**	cm/m2	1,4	2,0	2,0	2,3	2,3	2,6		2,6
**ÁTRIO ESQUERDO**		**NORMAL**		**DISCRETO**		**MODERADO**		**IMPORTANTE**	
**Diâmetro transversal 3C**	cm	2,3	3,8	3,8	4,5	4,5	5,3	>	5,3
**Indexado**	cm/m2	1,1	2,6	2,6	3,3	3,3	4,1	>	4,1
**Diâmetro longitudinal 4C**	cm	4,7	6,8	6,8	7,8	7,8	8,9	>	8,9
**Indexado**	cm/m2	2,4	3,9	3,9	4,6	4,6	5,4	>	5,4
**Área 2C**	cm2	15,0	27,0	27,0	33,0	33,0	39,0	>	39,0
**Indexado**	cm2/m2	8,0	14,0	14,0	17,0	17,0	20,0	>	20,0
**Área 4C**	cm2	15,0	27,0	27,0	33,0	33,0	39,0	>	39,0
**Indexado**	cm2/m2	8,0	14,0	14,0	17,0	17,0	20,0	>	20,0
**Volume (Biplanar)**	ml	35,0	101,0	101,0	134,0	134,0	167,0	>	167,0
**Indexado**	ml/m2	21,0	51,0	51,0	66,0	66,0	81,0	>	81,0
**OUTROS**		**NORMAL**		**DISCRETO**		**MODERADO**		**IMPORTANTE**	

**Diâmetro da artéria pulmonar**	cm	1,9	2,7	2,7	3,1	3,1	3,5	>	3,5
**Indexado**	cm/m2	1,0	1,5	1,5	1,7	1,7	1,9	>	1,9
**Diâmetro da raiz da aorta**	cm	2,9	4,1	4,1	4,7	4,7	5,3	>	5,3
**Indexado**	cm/m2	1,5	2,1	2,1	2,4	2,4	2,7	>	2,7
**Diâmetro aorta ascendente**	cm	2,6	4,1	4,1	4,8	4,8	5,6	>	5,6
**Indexado**	cm/m2	1,3	2,2	2,2	2,6	2,6	3,1	>	3,1
**VENTRÍCULO DIREITO**		**NORMAL**		**DISCRETO**		**MODERADO**		**IMPORTANTE**	
**Fração de ejeção**	%	44,0	68,5	44,0	33,5	33,5	23,0	>	23,0
**Volume diastólico final**	ml	92,0	212,0	212,0	272,0	272,0	332,0	>	332,0
**Indexado**	ml/m2	55,0	109,0	109,0	136,0	136,0	163,0	>	163,0
**Volume sistólico final**	ml	35,0	95,0	95,0	125,0	125,0	155,0	>	155,0
**Indexado**	ml/m2	20,5	47,5	47,5	61,0	61,0	74,5	>	74,5
**Volume sistólico**	ml	45,5	132,5	132,5	176,0	176,0	219,5	>	219,5
**Indexado**	ml/m2	27,0	69,0	69,0	90,0	90,0	111,0	>	111,0
**Débito cardíaco**	L/min	3,1	7,9	7,9	10,3	10,3	12,7	>	12,7
**Índice cardíaco**	L/min/m2	1,6	4,3	4,3	5,6	5,6	7,0	>	7,0
**ÁTRIO DIREITO**		**NORMAL**		**DISCRETO**		**MODERADO**		**IMPORTANTE**	
**Diâmetro transversal 4C**	cm	3,7	5,5	5,5	6,4	6,4	7,3	>	7,3
**Indexado**	cm/m2	1,9	3,1	3,1	3,7	3,7	4,3	>	4,3
**Diâmetro longitudinal 4C**	cm	4,2	6,3	6,3	7,3	7,3	8,4	>	8,4
**Indexado**	cm/m2	2,1	3,6	3,6	4,3	4,3	5,1	>	5,1
**Área 4C**	cm2	13,5	28,5	28,5	36,0	36,0	43,5	>	43,5
**Indexado**	cm2/m2	6,5	15,5	15,5	20,0	20,0	24,5	>	24,5
**Volume (Biplanar)**	ml	35,0	95,0	95,0	125,0	125,0	155,0	>	155,0
**Indexado**	ml/m2	20,0	56,0	56,0	74,0	74,0	92,0	>	92,0

Valores normais propostos para mulheres.
