# Supplementary material for: Valores de Normalidade para Ressonância Magnética Cardiovascular: Uma Revisão Analítico-Comparativa com Ênfase na População Brasileira
Source: Arq Bras Cardiol. 2026 Jul 21;123(6):e20250862. [Article in Portuguese] doi: 10.36660/abc.20250862 (PMC13399083; doi:10.36660/abc.20250862)
Supplement: Translation

# Translation: Normal Reference Values for Cardiovascular Magnetic Resonance: An Analytical–Comparative Review with Emphasis on the Brazilian Population

**DOI:** 10.36660/abc.20250862i

**Authors:** Jorge A. Torreão, Isabela Bispo Santos da Silva Costa, Antonildes N. Assunção-Jr, Ilan Gottlieb, Leonardo Sara da Silva, Tiago Senra, Tiago Augusto Magalhães, Marly Uellendahl, Hugo Bizetto Zampa, Gabriela Liberato, Walther Yoshiharu Ishikawa, Henrique Trad, Marcelo Hadlich, Otavio Coelho-Filho, Roberto Sasdelli, Juliano Lara Fernandes, Luiz Francisco Rodrigues de Ávila, Ibraim Masciarelli F. Pinto, Paulo R. Schvartzman, Carlos E. Rochitte

**Affiliations:** 1 São Paulo SP Brazil Instituto do Coração do Hospital das Clínicas da Faculdade de Medicina da Universidade de São Paulo, São Paulo, SP – Brazil; 2 Salvador BA Brazil Hospital Santa Izabel – Santa Casa da Bahia, Salvador, BA – Brazil; 3 São Paulo SP Brazil Diagnósticos da América S/A – DASA, São Paulo, SP – Brazil; 4 Salvador BA Brazil Rede D’Or Regional Bahia, Salvador, BA – Brazil; 5 São Paulo SP Brazil Universidade de São Paulo Instituto do Câncer do Estado de São Paulo, São Paulo, SP – Brazil; 6 Rio de Janeiro RJ Brazil Fonte Imagem Medicina Diagnóstica, Rio de Janeiro, RJ – Brazil; 7 Goiânia GO Brazil Clínica CDI PREMIUM, Goiânia, GO – Brazil; 8 São Paulo SP Brazil Instituto Dante Pazzanese de Cardiologia, São Paulo, SP – Brazil; 9 São Paulo SP Brazil Hospital Sírio Libanês, São Paulo, SP – Brazil; 10 Campinas SP Brazil Hospital Vera Cruz, Campinas, SP – Brazil; 11 Curitiba PR Brazil Complexo Hospital de Clínicas da Universidade Federal do Paraná (CHC-UFPR) – Cardiovascular CT/MR, Curitiba, PR – Brazil; 12 São Paulo SP Brazil Hospital do Coração (HCOR), São Paulo, SP – Brazil; 13 São Paulo SP Brazil Universidade Federal de São Paulo (UNIFESP), São Paulo, SP – Brazil; 14 São Paulo SP Brazil Hospitais São Luiz, Rede D’Or, São Paulo, SP – Brazil; 15 Ribeirão Preto SP Brazil Lotus Radiologia, Ribeirão Preto, SP – Brazil; 16 Rio de Janeiro RJ Brazil Instituto Nacional de Cardiologia, Rio de Janeiro, RJ – Brazil; 17 Campinas SP Brazil Universidade Estadual de Campinas, Campinas, SP – Brazil; 18 São Paulo SP Brazil Einstein Hospital Israelita, São Paulo, SP – Brazil; 19 Campinas SP Brazil Radiologia Clínica de Campinas, Campinas, SP – Brazil; 20 Campinas SP Brazil Instituto de Ensino e Pesquisa Jose Michel Kalaf, Campinas, SP – Brazil; 21 São Paulo SP Brazil Grupo Fleury, São Paulo, SP – Brazil; 22 Porto Alegre RS Brazil Hospital Moinhos de Vento, Porto Alegre, RS – Brazil

**Keywords:** Reference Values, Magnetic Resonance Imaging, Cardiovascular Disease

## Abstract

**Background:**

Cardiovascular magnetic resonance (CMR) is the reference standard for morphofunctional cardiac assessment. In Brazil, the application of international normal reference values faces challenges related to population admixture, technical heterogeneity among centers, and the absence of national standardization, all of which may affect diagnostic accuracy.

**Objectives:**

To compare normal reference values for morphofunctional parameters obtained by CMR between a multicenter international review and a Brazilian publication, discussing concordance, methodological differences, and implications for Brazilian clinical practice.

**Methods:**

An analytical–comparative review was performed using summarized data from an international reference-value review and a Brazilian study. Means and standard deviations of key ventricular variables were compared, with assessment of 95% CI overlap, Welch's t-test, and effect size (Hedges’ g). Standard-deviation–based cut-off points were proposed for grading abnormality.

**Results:**

Overall concordance between means was observed for most variables. Statistically significant differences occurred in a few parameters, mainly ventricular diameters and left ventricular mass, with small to moderate effect sizes. Discrepancies were largely attributed to methodological differences in measurement and indexation.

**Conclusions:**

International normal reference values may be used in Brazilian clinical practice, provided they are adjusted for body surface area and interpreted in light of local particularities. Standardization of protocols and national multicenter studies are needed to strengthen regional validation. Keywords: Normal Values, Cardiac Magnetic Resonance, Reference Interval.

## Introduction

Cardiac magnetic resonance (CMR) imaging has become the gold-standard method for morphofunctional assessment of the heart, offering a unique combination of high anatomical resolution, tissue characterization, and functional analysis.¹ Its ability to provide reproducible, three-dimensional measurements of ventricular volumes, ejection fraction, myocardial mass, and blood flow makes it indispensable for the diagnosis and follow-up of several cardiac conditions, including hypertrophic and arrhythmogenic cardiomyopathies, ischemic heart disease, congenital heart disease, myocarditis, amyloidosis, among others. In addition, CMR shows lower interobserver variability compared with echocardiography.^2^ It is the most reliable method for assessing left ventricular mass, which is crucial for the diagnosis of hypertrophy.^3,4^ Unlike computed tomography and nuclear imaging, CMR does not involve ionizing radiation and is safe for repeated evaluations, making it ideal for monitoring chronic diseases.^5^

CMR is an irreplaceable tool in modern cardiology, but its effectiveness in Brazil depends on the validation of locally derived normal reference values. The lack of standardization may lead to underdiagnosis or overdiagnosis, particularly in populations whose characteristics differ from those of international cohorts. Future multicenter Brazilian studies are essential to establish reliable national guidelines.

The Brazilian population is highly admixed, with European, African, and Indigenous genetic influences, which may affect cardiac parameters. Few national studies have defined normal CMR reference values, and many available investigations rely on small samples or are limited to specific regions. In 2013, a pioneering Brazilian study^6^ described reference values for cardiac parameters obtained by magnetic resonance imaging in a population of healthy Brazilian adults. The authors analyzed measures such as ventricular volumes, ejection fraction, and myocardial mass, providing relevant data for national clinical practice.

The study highlights particularities of the Brazilian population, which differs from international cohorts. Its findings contribute to the standardization of CMR interpretation in the Brazilian context, considering factors such as ethnic diversity and regional characteristics. The study represents a valuable foundation for future research and for the adaptation of cardiovascular diagnostic protocols in Brazil. It should be emphasized, however, that this study should not be interpreted as epidemiologically representative of the entire Brazilian population, but rather as the best national evidence currently available for comparative purposes.

In 2020, an important publication^7^ compiled normal reference values from multiple centers around the world and established normative CMR reference ranges for healthy adults and children. Owing to its multicenter, international, and multiethnic nature, this publication has become widely used as a reference for clinical evaluation and research in many countries. In 2025, these data were updated with the addition of new cohorts, making the dataset even more robust.^8^ The study provides parameters stratified by sex and age, including ventricular volumes, ejection fraction, and myocardial mass. It also highlights the importance of considering population and technical variations when interpreting examinations. Its findings are fundamental for improving diagnostic accuracy and standardizing CMR reporting, facilitating comparisons across different populations and medical centers. Notably, this international review incorporated data from the Brazilian publication, among other sources, to calculate some of the pooled averages.

Most imaging services in Brazil still rely on various international reference standards, which may not adequately reflect the local population. Additionally, centers use equipment from different manufacturers, with variations in imaging sequences, protocols, and acquisition parameters. The absence of unified national guidelines makes it difficult to compare results across institutions. CMR remains concentrated in major urban centers, with limited availability in peripheral regions. Given the scarcity of evidence on normal reference values in the country, this review aims to assess the feasibility of using internationally established reference values in Brazilian clinical practice.

Accordingly, the present study aims to compare the normal reference values proposed in an international review with those obtained in the national publication, to discuss the main methodological and clinical discrepancies observed, and to propose a standardization strategy tailored to the Brazilian context.

## Methods

This review follows an analytical-comparative and exploratory approach, using the normal reference values from the main international review on the topic^8^ and those from the Brazilian study.^6^ The means and standard deviations (SDs) of the main variables were analyzed, and dispersion levels were established based on the SDs reported in both studies. The degree of agreement between parameters was assessed, and potential sources of divergence were discussed (ethnic, methodological, and population-related factors). Normality cut-off points were also defined, emphasizing different degrees of abnormality (mild, moderate, and severe), using exploratory thresholds of 1.5, 3.0, and 4.5 standard deviations, respectively. It is important to note that access to the cohorts’ microdata was not possible, preventing analyses of true distribution patterns and multivariable adjustments.

### Study Methods

In the Brazilian study,^6^ an original investigation, participants underwent examinations using 1.5-Tesla scanners (Achieva, Philips Medical Systems, Best, The Netherlands; Signa CV/I, GE Medical Systems, Waukesha, WI; and Avanto, Siemens Medical Solutions, Erlangen, Germany). ECG-gated images were acquired during end-expiratory breath-hold (15-second respiratory pause) using dedicated cardiac coils. Image acquisition was performed with cine-SSFP (Steady-State Free Precession) sequences for the assessment of ventricular volumes and systolic function using the Simpson method, with short-axis slices covering the left ventricle from base to apex, and additional two- and four-chamber views.

Post-processing and analysis were performed by three experienced specialists using dedicated software (ViewForum, Philips Medical; and Argus, Siemens Medical Solutions) for quantification of ejection fraction (LVEF and RVEF), ventricular volumes (LVEDV, LVESV, RVEDV, RVESV), and left ventricular myocardial mass, with values indexed to body surface area (BSA). Endocardial and epicardial borders were manually traced in diastole and systole, excluding trabeculated myocardium and papillary muscles from the ventricular mass calculation.

The international publication^8^ is a review that conducted a PubMed search to identify studies reporting cardiovascular magnetic resonance (CMR) reference ranges. The summarized inclusion criteria were: 1. A minimum sample size of 40 individuals (or 40 per sex), considered the smallest acceptable number for calculating reference intervals using parametric methods; 2. Inclusion of only healthy cohorts, excluding individuals with conditions that could affect the analyzed parameters (e.g., hypertension, diabetes). Data from population-based studies (such as MESA and UK Biobank) were filtered to include only healthy subgroups; 3.Avoidance of duplicate cohorts appearing in multiple publications.

Studies were excluded if they used outdated CMR techniques, had missing data that could not be retrieved from authors, presented insufficient or inconsistent methodological descriptions, or employed methods not aligned with the Society for Cardiovascular Magnetic Resonance guidelines. The review aimed to standardize several CMR-derived variables, with broader scope than the Brazilian publication. Therefore, only the studies used for the variables analyzed in the present review are cited: left ventricular end-diastolic volume absolute and indexed (LVEDV and LVEDVi), left ventricular end-systolic volume absolute and indexed (LVESV and LVESVi), left ventricular ejection fraction (LVEF), left ventricular mass absolute and indexed (LVM and LVMi), left ventricular diastolic and systolic diameters (LVDD and LVSD), right ventricular end-diastolic volume absolute and indexed (RVEDV and RVEDVi), right ventricular end-systolic volume absolute and indexed (RVESV and RVESVi), and right ventricular ejection fraction (RVEF). It is noteworthy that the Brazilian publication is among the studies included in the international review.^3,6,9-26^

The method used to evaluate the left ventricle was cine-SSFP imaging on 1.5- or 3-Tesla scanners. Most studies used semi-automated software for left ventricular and right ventricular structural and functional analysis, applying the Simpson method on short-axis images, with papillary muscles included in the ventricular cavity volume.

### Statistical analysis

The comparison between the parameters of the two studies was conducted using three complementary statistical approaches. First, the overlap of the 95% confidence intervals (95% CI) of the means was assessed, using the standard error (SE) calculated by the formula SE = SD / √n. The absence of overlap between confidence intervals may be interpreted as suggestive of a statistically relevant difference.

Next, Welch's t-test for independent samples was applied, appropriate for situations with unequal variances and summarized data, in order to formally test the hypothesis of equality between means. Degrees of freedom were adjusted according to Welch's formula, and two-tailed p-values below 0.05 were considered indicative of statistical significance. To measure the practical relevance of the differences, the standardized effect size was calculated using Hedges’ g, which represents a correction of Cohen's d for samples of different or small sizes. The Hedges’ g statistic was interpreted according to Cohen's proposed thresholds, based on its absolute value: negligible/absent difference (< 0.2), small difference (0.2–0.5), moderate difference (0.5–0.8), or large difference (> 0.8).^27^ Statistical analyses were performed using the R language (version 4.4.3).

## Results

The cardiac morphofunctional variables obtained by CMR were compared between the two studies. The analysis was stratified by sex and conducted using the parameters shown in Figure 1.

**Figure 1:** Mean values of the analyzed variables.

Inspection of the 95% confidence intervals (95% CI) revealed substantial overlap for most parameters, suggesting a high degree of concordance between the sample results. The formal analysis using Welch's t-test indicated statistically significant differences (p < 0.05) in only a few variables. In addition to the statistical comparison, the clinical relevance of the differences was assessed using the standardized effect size (Hedges’ g), which provides an interpretable measure of the magnitude of the difference between groups.

Among men, significant differences were observed in absolute left ventricular mass (Hedges’ g: 0.44), indicating a small clinical difference, and in indexed mass (Hedges’ g: 0.59), as well as in left ventricular diameters (LVDD: 0.59 and LVSD: 0.66), the latter showing a moderate clinical effect. Among women, significant differences were found only in ventricular diameters (Hedges’ g: LVDD: 0.48 and LVSD: 0.59), indicating small and moderate clinical differences, respectively. For the remaining variables, differences were not statistically significant and showed small effect sizes (Hedges’ g < 0.5) (Table 1 and Central Illustration).

**Table 1:** Comparison of the international review values with the Brazilian publication

Publications		Macedo et al (6)					Kawel Bohem et al (8)					p	Hedges’ g	Clinical effect
Parameters	Sex	n	m	SD	95%CI		n	m	SD	95%CI				
LVEF	M	54	64.9	6.1	63.3	66.5	4854	64.0	7.0	63.8	64.1	0.28	0.128	Minimal
LVEDV	M	54	142.0	26.7	135.0	149.0	4867	143.0	31.0	142.1	143.8	0.78	0.032	Minimal
LVEDVi	M	54	74.2	13.1	70.7	77.7	4868	75.0	14.0	74.6	75.3	0.65	0.057	Minimal
LVESV	M	54	50.1	14.1	46.3	53.9	4857	52.0	18.0	51.4	52.5	0.33	0.105	Minimal
LVESVi	M	54	26.3	7.3	24.4	28.2	4847	28.0	8.0	27.7	28.2	0.09	0.212	Small
LVM	M	54	115.2	26.0	108.0	122.0	4859	105.0	23.0	104.3	105.6	0.005	0.442	Small
LVMi	M	54	59.8	11.7	56.7	62.9	4850	55.0	8.0	54.7	55.2	0.004	0.596	Moderate
LVEDV	M	54	4.9	0.5	4.8	5.0	500	5.2	0.5	5.1	5.2	0.0001	0.598	Moderate
LVESV	M	54	3.2	0.3	3.1	3.3	60	3.4	0.3	3.3	3.4	0.0005	0.662	Moderate
RVEF	M	54	57.1	7.1	55.2	59.0	5095	58.0	7.0	57.8	58.1	0.358	0.128	Minimal
RVEDV	M	54	149.0	33.8	140.0	158.0	5097	152.0	40.0	150.9	153.0	0.52	0.075	Minimal
RVEDVi	M	54	77.8	16.4	73.4	82.2	5095	82.0	18.0	81.5	82.4	0.066	0.233	Small
RVESV	M	54	64.1	19.3	59	69.2	5103	65.0	20.0	64.4	65.5	0.73	0.045	Minimal
RVESV i	M	54	33.4	9.0	31	35.8	5102	34.0	9.0	33.7	34.2	0.62	0.066	Minimal
LVEF	F	53	66.1	6.5	64.4	67.8	5209	66.0	6.0	65.8	66.1	0.91	0.016	Minimal
LVEDV	F	53	115.0	26.0	108.0	122.0	5213	113.0	23.0	112.3	113.6	0.579	0.086	Minimal
LVEDVi	F	53	68.5	16.8	64.0	73.0	5209	68.0	11.0	67.7	68.2	0.829	0.045	Minimal
LVESV	F	53	40.1	17.5	35.4	44.8	5203	39.0	13.0	38.6	39.3	0.65	0.084	Minimal
LVESVi	F	53	24.0	11.3	21.0	27.0	5200	23.0	6.0	22.8	23.1	0.52	0.164	Minimal
LVM	F	53	74.8	20.1	69.4	80.2	5203	74.0	14.0	73.6	74.3	0.773	0.056	Minimal
LVMi	F	53	44.6	11.7	41.5	47.7	5205	45.0	6.0	44.8	45.1	0.80	0.065	Minimal
LVEDV	F	53	4.6	0.5	4.5	4.7	672	4.8	0.4	4.7	4.8	0.008	0.487	Small
LVESV	F	53	2.8	0.6	2.6	3.0	60	3.1	0.4	2.9	3.2	0.002	0.591	Moderate
RVEF	F	53	61.0	7.8	58.9	63.1	5219	61.0	7.0	60.8	61.1	1.0	0.0	Minimal
RVEDV	F	53	115.0	26.0	108.0	122.0	5219	115.0	29.0	114.2	115.7	1.0	0.0	Minimal
RVEDVi	F	53	68.6	15.2	64.5	72.7	5221	71.0	14.0	70.6	71.3	0.25	0.171	Minimal
RVESV	F	53	45.4	14.3	41.6	49.2	5216	46.0	15.0	45.5	46.4	0.76	0.040	Minimal
RVESV i	F	53	27.1	8.5	24.8	29.4	5214	28.0	8.0	27.7	28.2	0.44	0.112	Minimal

LVEDV: left ventricular end-diastolic volume; LVEDVi: left ventricular end-diastolic volume (indexed); LVESV and LVESVi: absolute and indexed left ventricular end-systolic volume, respectively; LVM and LVMi: left ventricular mass (absolute and indexed, respectively); LFEV: left ventricular ejection fraction; LVDD left ventricular diastolic diameter; LVSD: left ventricular systolic diameter; RVEF: right ventricular ejection fraction; RVEDV and RVEDVi: right ventricular end-diastolic volume (absolute and indexed, respectively); RVESV and RVESVi: right ventricular end-systolic volume (absolute and indexed, respectively). Sample size (n); mean (m); standard deviation (SD); confidence interval (CI); women (W); men (M). Significant p-value < 0.05, Welch's t-test. Clinical effect of CI differences assessed using Hedges’ g. Differences between means were classified as negligible/absent (< 0.3), small (0.3–0.5), moderate (0.5–0.8), or large (> 0.8).

After confirming the negligible or small divergence between the means of the two publications, values were extracted from the means and standard deviations (1/1.5/2/2.5/3/3.5/4/4.5/5/5.5/6 SD) of the main variables of interest identified in the international review, followed by an analysis of cut-off points emphasizing different degrees of abnormality (mild, moderate, and severe). After careful evaluation, the thresholds of 1.5, 3, and 4.5 standard deviations were selected as cut-off points, respectively. Values for women are presented in Table 2 and for men in Table 3. Finally, a table proposing a standardization strategy was developed and is presented in Supplementary Tables S1 and S2.

**Table 2:** Mean and standard deviation for female patients (8)

						STANDARD DEVIATION											
LEFT VENTRICLE		m	dp	LI	LS		1	1.5	2	2.5	3	3.5	4	4.5	5	5.5	6
LVEF	%	66.0	6.0	57.0	75.0		60.0	57.0	54.0	51.0	48.0	45.0	42.0	39.0	36.0	33.0	30.0
LVEDV	mL	113.0	23.0	78.5	147.5		136.0	147.5	159.0	170.5	182.0	193.5	205.0	216.5	228.0	239.5	251.0
LVEDVi	mL/m^2^	68.0	11.0	51.5	84.5		79.0	84.5	90.0	95.5	101.0	106.5	112.0	117.5	123.0	128.5	134.0
LVESV	mL	39.0	13.0	19.5	58.5		52.0	58.5	65.0	71.5	78.0	84.5	91.0	97.5	104.0	110.5	117.0
LVESV i	mL/m^2^	23.0	6.0	14.0	32.0		29.0	32.0	35.0	38.0	41.0	44.0	47.0	50.0	53.0	56.0	59.0
LVSV	mL	74.0	14.0	53.0	95.0		88.0	95.0	102.0	109.0	116.0	123.0	130.0	137.0	144.0	151.0	158.0
LVSVi	mL/m^2^	45.0	6.0	36.0	54.0		51.0	54.0	57.0	60.0	63.0	66.0	69.0	72.0	75.0	78.0	81.0
LVM	g	74.0	14.0	53.0	95.0		88.0	95.0	102.0	109.0	116.0	123.0	130.0	137.0	144.0	151.0	158.0
LVMi	g/m^2^	45.0	6.0	36.0	54.0		51.0	54.0	57.0	60.0	63.0	66.0	69.0	72.0	75.0	78.0	81.0
LVCO	L/min	4.9	1.1	3.3	6.6		6.0	6.6	7.1	7.7	8.2	8.8	9.3	9.9	10.4	11.0	11.5
LVCOi	L/min/m^2^	3.0	0.6	2.1	3.9		3.6	3.9	4.2	4.5	4.8	5.1	5.4	5.7	6.0	6.3	6.6
Anteroseptal	cm	0.7	0.1	0.5	1.0		0.9	1.0	1.0	1.1	1.2	1.2	1.3	1.4	1.4	1.5	1.6
Inferolateral	cm	0.6	0.1	0.5	0.8		0.8	0.8	0.9	0.9	1.0	1.1	1.1	1.2	1.2	1.3	1.4
LVEDD	cm	4.8	0.4	4.2	5.4		5.2	5.4	5.6	5.8	6.0	6.2	6.4	6.6	6.8	7.0	7.2
LVEDDi	cm/m^2^	3.2	0.4	2.6	3.8		3.6	3.8	4.0	4.2	4.4	4.6	4.8	5.0	5.2	5.4	5.6
LVESD	cm	3.1	0.4	2.5	3.7		3.5	3.7	3.9	4.1	4.3	4.5	4.7	4.9	5.1	5.3	5.5
LVESDi	cm/m^2^	1.7	0.4	1.1	2.3		2.1	2.3	2.5	2.7	2.9	3.1	3.3	3.5	3.7	3.9	4.1
**LEFT ATRIUM**																	
Transverse in 3CH	cm	3.0	1.0	1.5	4.5		4.0	4.5	5.0	5.5	6.0	6.5	7.0	7.5	8.0	8.5	9.0
Indexed	cm/m^2^	2.0	1.0	0.5	3.5		3.0	3.5	4.0	4.5	5.0	5.5	6.0	6.5	7.0	7.5	8.0
Longitudinal in 4CH	cm	5.4	0.7	4.4	6.5		6.1	6.5	6.8	7.2	7.5	7.9	8.2	8.6	8.9	9.3	9.6
Indexed	cm/m^2^	3.4	0.6	2.5	4.3		4.0	4.3	4.6	4.9	5.2	5.5	5.8	6.1	6.4	6.7	7.0
Area in 2CH	cm^2^	18.0	4.0	12.0	24.0		22.0	24.0	26.0	28.0	30.0	32.0	34.0	36.0	38.0	40.0	42.0
Indexed	cm^2^/m^2^	11.0	3.0	6.5	15.5		191.0	182.0	173.0	164.0	155.0	146.0	137.0	128.0	119.0	110.0	101.0
Ara in 4CH	cm^2^	19.0	4.0	13.0	25.0		23.0	25.0	27.0	29.0	31.0	33.0	35.0	37.0	39.0	41.0	43.0
Indexed	cm^2^/m^2^	11.0	2.0	8.0	14.0		13.0	14.0	15.0	16.0	17.0	18.0	19.0	20.0	21.0	22.0	23.0
Volume (Biplanar)	mL	60.0	17.0	34.5	85.5		77.0	85.5	94.0	102.5	111.0	119.5	128.0	136.5	145.0	153.5	162.0
Indexed	mL/m^2^	36.0	9.0	22.5	49.5		45.0	49.5	54.0	58.5	63.0	67.5	72.0	76.5	81.0	85.5	90.0
**OTHERS**																	
Pulmonary artery	cm	2.2	0.3	1.8	2.5		2.4	2.5	2.7	2.8	2.9	3.0	3.2	3.3	3.4	3.5	3.7
Indexed	cm/m^2^	1.3	0.2	1.1	1.6		1.5	1.6	1.7	1.7	1.8	1.9	2.0	2.1	2.1	2.2	2.3
Aortic root	cm	3.1	0.3	2.6	3.6		3.4	3.6	3.7	3.9	4.1	4.2	4.4	4.6	4.7	4.9	5.1
Indexed	cm/m^2^	1.8	0.2	1.5	2.1		2.0	2.1	2.2	2.3	2.4	2.5	2.6	2.7	2.8	2.9	3.0
Ascending aorta	cm	3.0	0.6	2.1	3.9		3.6	3.9	4.2	4.5	4.8	5.1	5.4	5.7	6.0	6.3	6.6
Indexed	cm/m^2^	1.8	0.3	1.4	2.3		2.1	2.3	2.4	2.6	2.7	2.9	3.0	3.2	3.3	3.5	3.6
**RIGHT VENTRICLE**																	
RVEF	%	61.0	7.0	50.5	71.5		54.0	50.5	47.0	43.5	40.0	36.5	33.0	29.5	26.0	22.5	19.0
RVEDV	mL	115.0	69.0	11.5	218.5		184.0	218.5	253.0	287.5	322.0	356.5	391.0	425.5	460.0	494.5	529.0
RVEDVi	mL/m^2^	71.0	14.0	50.0	92.0		85.0	92.0	99.0	106.0	113.0	120.0	127.0	134.0	141.0	148.0	155.0
RVESV	mL	46.0	15.0	23.5	68.5		61.0	68.5	76.0	83.5	91.0	98.5	106.0	113.5	121.0	128.5	136.0
RVESVi	mL/m^2^	28.0	8.0	16.0	40.0		36.0	40.0	44.0	48.0	52.0	56.0	60.0	64.0	68.0	72.0	76.0
RVSV	mL	71.0	20.0	41.0	101.0		91.0	101.0	111.0	121.0	131.0	141.0	151.0	161.0	171.0	181.0	191.0
RVSVi	mL/m^2^	45.0	10.0	30.0	60.0		55.0	60.0	65.0	70.0	75.0	80.0	85.0	90.0	95.0	100.0	105.0
RVCO	L/min	4.6	1.2	2.8	6.4		5.8	6.4	7.0	7.6	8.2	8.8	9.4	10.0	10.6	11.2	11.8
RVCOi	L/min/m^2^	2.7	0.7	1.7	3.8		3.4	3.8	4.1	4.5	4.8	5.2	5.5	5.9	6.2	6.6	6.9
**RIGHT ATRIUM**																	
Transverse in 4CH	cm	4.1	0.7	3.1	5.2		4.8	5.2	5.5	5.9	6.2	6.6	6.9	7.3	7.6	8.0	8.3
Indexed	cm/m^2^	2.6	0.4	2.0	3.2		3.0	3.2	3.4	3.6	3.8	4.0	4.2	4.4	4.6	4.8	5.0
Longitudinal in 4CH	cm	4.9	0.7	3.9	6.0		5.6	6.0	6.3	6.7	7.0	7.4	7.7	8.1	8.4	8.8	9.1
Indexed	cm/m^2^	3.1	0.5	2.4	3.9		3.6	31.6	30.1	28.6	27.1	25.6	24.1	22.6	21.1	19.6	18.1
Area in 4Ch	cm^2^	18.0	0.4	17.4	18.6		18.4	18.6	18.8	19.0	19.2	19.4	19.6	19.8	20.0	20.2	20.4
Indexed	cm^2^/m^2^	11.0	0.2	10.7	11.3		11.2	11.3	11.4	11.5	11.6	11.7	11.8	11.9	12.0	12.1	12.2
Volume (Biplane)	mL	53.0	14.0	32.0	74.0		23.0	74.0	81.0	88.0	95.0	102.0	109.0	116.0	123.0	130.0	137.0
Indexed	mL/m^2^	35.0	10.0	20.0	50.0		38.2	50.0	55.0	60.0	65.0	70.0	75.0	80.0	85.0	90.0	95.0

LVEDV: left ventricular end-diastolic volume; LVEDVi: left ventricular end-diastolic volume (indexed); LVESV and LVESVi: absolute and indexed left ventricular end-systolic volume, respectively; LVM and LVMi: left ventricular mass (absolute and indexed, respectively); LFEV: left ventricular ejection fraction; LVDD left ventricular diastolic diameter; LVSD: left ventricular systolic diameter; RVEF: right ventricular ejection fraction; RVEDV and RVEDVi: right ventricular end-diastolic volume (absolute and indexed, respectively); RVESV and RVESVi: right ventricular end-systolic volume (absolute and indexed, respectively). Mean (m); standard deviation (SD); four-chamber view (4CH); three-chamber view (3CH)

**Table 3:** Mean and standard deviation for male patients (8)

							STANDARD DEVIATION										
LEFT VENTRICLE		m	dp	LI	LS		1	1.5	2	2.5	3	3.5	4	4.5	5	5.5	6
LVEF	%	64.0	7.0	53.5	74.5	57.0	53.5	50.0	46.5	43.0	39.5	36.0	32.5	29.0	25.5	22.0	64.0
LVEDV	mL	143.0	31.0	96.5	189.5	174.0	189.5	205.0	220.5	236.0	251.5	267.0	282.5	298.0	313.5	329.0	143.0
LVEDVi	mL/m^2^	75.0	14.0	54.0	96.0	89.0	96.0	103.0	110.0	117.0	124.0	131.0	138.0	145.0	152.0	159.0	75.0
LVESV	mL	52.0	18.0	25.0	79.0	70.0	79.0	88.0	97.0	106.0	115.0	124.0	133.0	142.0	151.0	160.0	52.0
LVESVi	mL/m^2^	28.0	8.0	16.0	40.0	36.0	40.0	44.0	48.0	52.0	56.0	60.0	64.0	68.0	72.0	76.0	28.0
LVSV	mL	91.0	19.0	62.5	119.5	110.0	119.5	129.0	138.5	148.0	157.5	167.0	176.5	186.0	195.5	205.0	91.0
LVSVi	mL/m^2^	47.0	9.0	33.5	60.5	56.0	60.5	65.0	69.5	74.0	78.5	83.0	87.5	92.0	96.5	101.0	47.0
LVM	g	105.0	23.0	70.5	139.5	128.0	139.5	151.0	162.5	174.0	185.5	197.0	208.5	220.0	231.5	243.0	105.0
LVMi	g/m^2^	55.0	8.0	43.0	67.0	63.0	67.0	71.0	75.0	79.0	83.0	87.0	91.0	95.0	99.0	103.0	55.0
LVCO	L/min	6.1	1.3	4.2	8.1	7.4	8.1	8.7	9.4	10.0	10.7	11.3	12.0	12.6	13.3	13.9	6.1
LVCOI	L/min/m^2^	3.2	0.6	2.3	4.1	3.8	4.1	4.4	4.7	5.0	5.3	5.6	5.9	6.2	6.5	6.8	3.2
Anteroseptal	cm	0.9	0.2	0.7	1.1	1.1	1.1	1.2	1.3	1.4	1.5	1.5	1.6	1.7	1.8	1.9	0.9
Inferolateral	cm	0.8	0.1	0.6	1.0	0.9	1.0	1.1	1.1	1.2	1.3	1.3	1.4	1.5	1.6	1.6	0.8
LVEDD	cm	5.2	0.5	4.5	6.0	5.7	6.0	6.2	6.5	6.7	7.0	7.2	7.5	7.7	8.0	8.2	5.2
LVEDDi	cm/m^2^	2.6	0.3	2.2	3.1	2.9	3.1	3.2	3.4	3.5	3.7	3.8	4.0	4.1	4.3	4.4	2.6
LVESD	cm	3.4	0.3	3.0	3.9	3.7	3.9	4.0	4.2	4.3	4.5	4.6	4.8	4.9	5.1	5.2	3.4
LVESDi	cm/m^2^	1.7	0.2	1.4	2.0	1.9	2.0	2.1	2.2	2.3	2.4	2.5	2.6	2.7	2.8	2.9	1.7
**LEFT ATRIUM**																	
Transverse in 3CH	cm	3.0	0.5	2.3	3.8	3.5	3.8	4.0	4.3	4.5	4.8	5.0	5.3	5.5	5.8	6.0	3.0
Indexed	cm/m^2^	1.8	0.5	1.1	2.6	2.3	2.6	2.8	3.1	3.3	3.6	3.8	4.1	4.3	4.6	4.8	1.8
Longitudinal in 4CH	cm	5.7	0.7	4.7	6.8	6.4	6.8	7.1	7.5	7.8	8.2	8.5	8.9	9.2	9.6	9.9	5.7
Indexed	cm/m^2^	3.1	0.5	2.4	3.9	3.6	3.9	4.1	4.4	4.6	4.9	5.1	5.4	5.6	5.9	6.1	3.1
Area in2CH	cm^2^	21.0	4.0	15.0	27.0	25.0	27.0	29.0	31.0	33.0	35.0	37.0	39.0	41.0	43.0	45.0	21.0
Indexed	cm^2^/m^2^	11.0	2.0	8.0	14.0	13.0	14.0	15.0	16.0	17.0	18.0	19.0	20.0	21.0	22.0	23.0	11.0
Area in 4Ch	cm^2^	21.0	4.0	15.0	27.0	25.0	27.0	29.0	31.0	33.0	35.0	37.0	39.0	41.0	43.0	45.0	21.0
Indexed	cm^2^/m^2^	11.0	2.0	8.0	14.0	13.0	14.0	15.0	16.0	17.0	18.0	19.0	20.0	21.0	22.0	23.0	11.0
Volume (Biplane)	mL	68.0	22.0	35.0	101.0	90.0	101.0	112.0	123.0	134.0	145.0	156.0	167.0	178.0	189.0	200.0	68.0
Indexed	mL/m^2^	36.0	10.0	21.0	51.0	46.0	51.0	56.0	61.0	66.0	71.0	76.0	81.0	86.0	91.0	96.0	36.0
**OTHERS**																	
Pulmonary artery	cm	2.3	0.3	1.9	2.7	2.6	2.7	2.8	3.0	3.1	3.2	3.4	3.5	3.6	3.7	3.9	2.3
Indexed	cm/m^2^	1.2	0.1	1.0	1.5	1.4	1.5	1.5	1.6	1.7	1.7	1.8	1.9	1.9	2.0	2.1	1.2
Aortic root	cm	3.5	0.4	2.9	4.1	3.9	4.1	4.3	4.5	4.7	4.9	5.1	5.3	5.5	5.7	5.9	3.5
Indexed	cm/m^2^	1.8	0.2	1.5	2.1	2.0	2.1	2.2	2.3	2.4	2.5	2.6	2.7	2.8	2.9	3.0	1.8
Ascending aorta	cm	3.3	0.5	2.6	4.1	3.8	4.1	4.3	4.6	4.8	5.1	5.3	5.6	5.8	6.1	6.3	3.3
Indexed	cm/m^2^	1.7	0.3	1.3	2.2	2.0	2.2	2.3	2.5	2.6	2.8	2.9	3.1	3.2	3.4	3.5	1.7
**RIGHT VENTRICLE**																	
RVEF	%	58.0	7.0	47.5	68.5	51.0	47.5	44.0	40.5	37.0	33.5	30.0	26.5	23.0	19.5	16.0	58.0
RVEDV	mL	152.0	40.0	92.0	212.0	192.0	212.0	232.0	252.0	272.0	292.0	312.0	332.0	352.0	372.0	392.0	152.0
RVEDVi	mL/m^2^	82.0	18.0	55.0	109.0	100.0	109.0	118.0	127.0	136.0	145.0	154.0	163.0	172.0	181.0	190.0	82.0
RVESV	mL	65.0	20.0	35.0	95.0	85.0	95.0	105.0	115.0	125.0	135.0	145.0	155.0	165.0	175.0	185.0	65.0
RVESVi	mL/m^2^	34.0	9.0	20.5	47.5	43.0	47.5	52.0	56.5	61.0	65.5	70.0	74.5	79.0	83.5	88.0	34.0
RVSV	mL	89.0	29.0	45.5	132.5	118.0	132.5	147.0	161.5	176.0	190.5	205.0	219.5	234.0	248.5	263.0	89.0
RVSVi	ml/m^2^	48.0	14.0	27.0	69.0	62.0	69.0	76.0	83.0	90.0	97.0	104.0	111.0	118.0	125.0	132.0	48.0
RVCO	L/min	5.5	1.6	3.1	7.9	7.1	7.9	8.7	9.5	10.3	11.1	11.9	12.7	13.5	14.3	15.1	5.5
RVCOi	L/min/m^2^	2.9	0.9	1.6	4.3	3.8	4.3	4.7	5.2	5.6	6.1	6.5	7.0	7.4	7.9	8.3	2.9
**RIGHT ATRIUM**																	
Transverse in 4CH	cm	4.6	0.6	3.7	5.5	5.2	5.5	5.8	6.1	6.4	6.7	7.0	7.3	7.6	7.9	8.2	4.6
Indexed	cm/m^2^	2.5	0.4	1.9	3.1	2.9	3.1	3.3	3.5	3.7	3.9	4.1	4.3	4.5	4.7	4.9	2.5
Longitudinal in 4CH	cm	5.2	0.7	4.2	6.3	5.9	6.3	6.6	7.0	7.3	7.7	8.0	8.4	8.7	9.1	9.4	5.2
Indexed	cm/m^2^	2.8	0.5	2.1	3.6	3.3	29.6	27.8	26.1	24.3	22.6	20.8	19.1	17.3	15.6	13.8	2.8
Area in 4Ch	cm^2^	21.0	5.0	13.5	28.5	26.0	28.5	31.0	33.5	36.0	38.5	41.0	43.5	46.0	48.5	51.0	21.0
Indexed	cm^2^/m^2^	11.0	3.0	6.5	15.5	14.0	15.5	17.0	18.5	20.0	21.5	23.0	24.5	26.0	27.5	29.0	11.0
Volume (Biplane)	ml	65.0	20.0	35.0	95.0	182.0	95.0	105.0	115.0	125.0	135.0	145.0	155.0	165.0	175.0	185.0	65.0
Indexed	ml/m^2^	38.0	12.0	20.0	56.0	533.0	56.0	62.0	68.0	74.0	80.0	86.0	92.0	98.0	104.0	110.0	38.0

LVEDV: left ventricular end-diastolic volume; LVEDVi: left ventricular end-diastolic volume (indexed); LVESV and LVESVi: absolute and indexed left ventricular end-systolic volume, respectively; LVM and LVMi: left ventricular mass (absolute and indexed, respectively); LFEV: left ventricular ejection fraction; LVDD left ventricular diastolic diameter; LVSD: left ventricular systolic diameter; RVEF: right ventricular ejection fraction; RVEDV and RVEDVi: right ventricular end-diastolic volume (absolute and indexed, respectively); RVESV and RVESVi: right ventricular end-systolic volume (absolute and indexed, respectively). Mean (m); standard deviation (SD); four-chamber view (4CH); three-chamber view (3CH).

## Discussion

The use of the reference values established by the international review^8^ in Brazilian clinical practice warrants careful consideration. This publication demonstrated overall agreement between the population means reported in the international review and those obtained in the Brazilian study,^6^ paving the way for the standardization of CMR imaging in the country. Such an approach would offer significant advantages, including the immediate adoption of internationally validated parameters, harmonization with global reference centers, and facilitation of multicenter studies.

It is important to emphasize that the statistical comparison using Welch's test was performed to ensure the scientific rigor of the publication; however, for assessing clinical relevance, Hedges’ g was used. This metric helps determine whether a difference between groups is not only statistically significant but also clinically meaningful, providing an estimate of the magnitude of the effect. According to conventional thresholds, Hedges’ g values indicate negligible/absent(< 0.2), small (< 0.5), moderate (0.5–0.8), or large (> 0.8) clinical differences.

Furthermore, in cases where a moderate divergence was observed between datasets – indexed mass, LVDD and LVSD in men; LVESVi in women – it is important to note that different methodologies were used in the two publications for measuring left ventricular diameters. In the Brazilian study, measurements were obtained from the four-chamber long-axis view, a technique now largely outdated, whereas the international review used short-axis measurements. Regarding indexed mass, some variability may arise due to the complex morphology of the left atrial-ventricular junction, which can make it difficult to delineate the left ventricular myocardium from the atrial wall. As for RVEDVi, which showed a small clinical difference without statistical significance, variability is expected due to the complex morphology and compliance of the right ventricle. Normative RV volume values may differ by as much as 20-30% depending on methodological variations and population characteristics.^3^

To categorize the dispersion of cardiac ventricular volumes in a normal sample (Gaussian distribution), the most used and statistically robust methodology is based on standard deviations (SDs) from the mean, which allows an objective classification of abnormalities (mild, moderate, severe). It should be emphasized that, due to the absence of a formal standard for this specific classification, an exploratory evaluation was performed, followed by a proposed suggestion. Among the options considered, the following can be highlighted:

First option: 1, 2, 3 SD – Advantage: simple and aligned with the empirical rule (68–95–99.7% of the population within 1, 2, 3 SD). Limitation: too broad for mild abnormalities (1 SD already includes ∼16% of the extremes); second option: 1.5, 3, 4.5 SD: Advantage: expands the "normal" range (1.5 SD includes ∼86% of the population), reducing false positives for mild abnormalities. Limitation: wider spacing between thresholds may underestimate gradations; third option: 2, 3, 4 SD – Advantage: more stringent definition of "mild" abnormality (2 SD ≈ 5% of extremes), useful for detecting clinically relevant deviations. Limitation: may be overly restrictive when normal variability is high.

After careful consideration, the second option (1.5, 3, and 4.5 SD) was selected, with the primary objective of avoiding overdiagnosis of mild abnormalities (1.5 SD is less sensitive than 1 SD) and prioritizing specificity (e.g., classifying as "moderate" only values above 3 SD, requiring a greater deviation from the mean). Thus, defining 1.5 SD for "mild" abnormality balances sensitivity and specificity; 3 SD for "moderate" abnormality indicates clearly abnormal deviations; and 4.5 SD for "severe" abnormality captures rare and marked cases.^3^ The exception was the classification of LVEF for men and women, which required minor adjustments to better align with values already established in the literature and clinical practice. It is important to reinforce that these cut-off points are not included in SCMR recommendations and should not be interpreted as validated thresholds.

The study by Petersen et al.^16^ demonstrated that reference values obtained in Caucasian populations showed good agreement with other populations when appropriately indexed to BSA. These findings suggest that, with proper methodological considerations, the application of international reference values to our population may be clinically valid.

Regardless of the degree of concordance observed, the results of this analysis may contribute to the ongoing discussion on the standardization of CMR imaging in Brazil. The practical implications of potentially adopting these reference values are substantial. From a clinical standpoint, it would provide greater confidence in exam interpretation and improve comparability across institutions. From a healthcare management perspective, it could reduce costs by avoiding the need for extensive local studies to establish population-specific parameters. For scientific research, it would facilitate integration with international studies and participation in global consortia.

Furthermore, these results may support Brazilian scientific societies in developing national guidelines, helping balance the adoption of international standards with the specific characteristics of our population.

### Limitations

This study has several limitations that should be considered when interpreting the results. The Brazilian study likely presents selection bias, with a smaller and regionally restricted sample composed predominantly of individuals evaluated in centers located in the Southeast. This limitation raises concerns about the applicability of these parameters to other regions of the country, particularly the North and Northeast, where population and environmental characteristics may differ substantially. Another relevant point is the regional variation in body mass index (BMI) within the Brazilian population, with obesity prevalence ranging from 9.9% (Northeast) to 15.9% (South),^28^ underscoring the importance of future nationwide studies. The international review, in contrast, includes adjustments and stratifications that are not available in the Brazilian study.

This study does not aim to formally validate the international reference values for the Brazilian population. It is an analysis of overall concordance between summarized means, without access to microdata or individual adjustments for age, BSA, or ethnicity. For this reason, no adjustments were made for demographic factors (age, height, BSA, ethnicity). The comparison between cohorts does not account for potential differences in age distribution, ethnic composition, or BSA. The marked admixture of the Brazilian population contrasts with predominantly European or North American cohorts, which may affect the extrapolation of findings. These limitations highlight the need for caution in interpreting and applying the results.

It was not possible to access the cohorts’ microdata; without individual-level information, the comparison relies on strong assumptions (such as normality and symmetry) and prevents analyses of the true distribution and multivariable adjustments, which may reduce the reliability of statistical tests and reference interval calculations. The inability to adjust for age, BSA, ethnicity, additional stratifications, or to explore the actual distribution limits stronger conclusions regarding population equivalence.

Regarding technical variability, the multicenter nature of the publications introduces potential impacts on analysis and comparison. The two studies present relevant methodological differences – equipment, protocols, measurement planes, software, and criteria for inclusion/exclusion of papillary muscles. Both use cine-SSFP as the primary technique for morphofunctional assessment, apply the Simpson method for volumetric quantification, and follow recommendations aligned with SCMR guidelines for ventricular volume and function acquisition and analysis. However, these differences limit direct comparisons and any inference of population equivalence. Therefore, the exploratory, analytical-comparative nature of this study must be emphasized, as it is subject to methodological biases and does not constitute formal validation between cohorts.

Additionally, the operational reality of imaging services in Brazil shows considerable heterogeneity in equipment and protocols, a factor that may compromise the uniformity of results.

### Future Perspectives

It is recommended that the adoption of the international review's reference values in Brazilian clinical practice be accompanied by complementary initiatives. Future studies with large Brazilian cohorts, representative of different regions of the country and of both public and private healthcare systems, would be highly desirable. Special attention should be given to specific population subgroups, such as distinct age ranges, athletes, individuals with obesity, or particular ethnic groups. Ideally, such studies should provide access to microdata, allow adjusted statistical models, and enable weighted resampling. Likewise, standardizing CMR acquisition and analysis protocols across Brazilian centers, in accordance with established guidelines, is essential to ensure the reliability and comparability of results.

## Conclusions

In this context, it seems reasonable to state that there is overall concordance between population means, with sufficient evidence to suggest that the reference values from the international review may be applied to the Brazilian population, provided that appropriate precautions are taken. This strategy represents a viable and potentially beneficial alternative, as long as it is accompanied by careful evaluation of local particularities, corresponding age ranges, and the examination methodologies used. Such an approach may constitute an important step forward for cardiovascular imaging in Brazil, enabling greater standardization of examinations and improved integration with the international scientific community, without disregarding the specific characteristics of the Brazilian population.

However, it is important to emphasize that these results should be interpreted with caution when extrapolated to clinical decision-making, particularly in borderline areas between normality and pathology. Additionally, the proposed cut-off points (1.5 / 3.0 / 4.5 SD) should be understood as a pragmatic, exploratory suggestion intended for clinical application, rather than as normative recommendations.

## Data Availability

The underlying content of the research text is contained within the manuscript.

## *Supplemental Materials

Supplemental Materials For additional information, please click here.
